# Future body mass index modelling based on macronutrient profiles and physical activity

**DOI:** 10.1186/1742-4682-9-43

**Published:** 2012-10-29

**Authors:** David K Cundiff, Nikunj Raghuvanshi

**Affiliations:** 1 Independent researcher, 333 Orizaba Avenue, Long Beach, CA, 90814, USA; 2 Researcher, Microsoft Research (this article is not job-related), 8935 160th Ave NE, Apt B415, Redmond, WA, 98052, USA

**Keywords:** Obesity, Obesity epidemic, Obesity prevention, Macronutrient profiling, Body mass index, Type 1 diabetes

## Abstract

**Background:**

An accurate system of determining the relationship of macronutrient profiles of foods and beverages to the long-term weight impacts of foods is necessary for evidence-based, unbiased front-of-the-package food labels.

**Methods:**

Data sets on diet, physical activity, and BMI came from the Food and Agriculture Organization (FAO), the World Health Organization (WHO), the Diabetes Control and Complications Trial (DCCT), and Epidemiology Diabetes Intervention and Complications (EDIC). To predict future BMI of individuals, multiple regression derived FAO/WHO and DCCT/EDIC formulas related macronutrient profiles and physical activity (independent variables) to BMI change/year (dependent variable). Similar formulas without physical activity related macronutrient profiles of individual foods and beverages to four-year weight impacts of those items and compared those forecasts to published food group profiling estimates from three large prospective studies by Harvard nutritional epidemiologists.

**Results:**

FAO/WHO food and beverage formula: four-year weight impact (pounds)=(0.07710 alcohol g+11.95 (381.7+carbohydrates g per serving)*4/(2,613+kilocalories per serving)–304.9 (30.38+dietary fiber g per serving)/(2,613+kilocalories per serving)+19.73 (84.44+total fat g)*9/(2,613+kilocalories per serving)–68.57 (20.45+PUFA g per serving)*9/(2,613+kilocalories per serving))*2.941–12.78 (n=334, R^2^=0.29, *P *< 0.0001). DCCT/EDIC formula for four-year weight impact (pounds)=(0.898 (102.2+protein g per serving)*4/(2,297+kilocalories per serving)+1.063 (264.2+carbohydrates g per serving)*4/(2,297+ kilocalories per serving)–13.19 (24.29+dietary fiber g per serving)/ (2,297+kilocalories per serving)+ 0.973 (74.59+(total fat g per serving–PUFA g per serving)*9/(2,297+kilocalories per serving))*85.82–68.11 (n=1,055, R^2^=0.03, *P *< 0.0001). (FAO/WHO+ DCCT/EDIC formula forecasts averaged correlated strongly with published food group profiling findings except for potatoes and dairy foods (n=12, r=0.85, *P = *0.0004). Formula predictions did not correlate with food group profiling findings for potatoes and dairy products (n=10, r= −0.33 *P*=0.36). A formula based diet and exercise analysis tool is available to researchers and individuals: http://thehealtheconomy.com/healthTool/.

**Conclusions:**

Two multiple regression derived formulas from dissimilar databases produced markedly similar estimates of future BMI for 1,055 individuals with type 1 diabetes and female and male cohorts from 167 countries. These formulas predicted the long-term weight impacts of foods and beverages, closely corresponding with most food group profiling estimates from three other databases. If discrepancies with potatoes and dairy products can be resolved, these formulas present a potential basis for a front-of-the-package weight impact rating system.

## Introduction

Previous mathematical modelling approaches to predict weight change have largely looked at short term results of changes of energy intake and expenditure [1,2]. As noted in one article using USA data, "A small persistent average daily energy imbalance gap between intake and expenditure of about 30 kJ per day underlies the observed average weight gain "[3]. The effect of the macronutrient profile, independent of daily energy intake, has been given less attention. Many researchers have characterized the etiology of the worldwide obesity epidemic as much more complex that simply an imbalance of calories in versus calories out. There are both complex economic causes and consequences of obesity [4]. For example, the food industry in Europe invested €1-billion in a campaign to block evidence-based health warnings on food [5]. Producing refined and processed convenience foods, high in sugar and saturated fats, is lucrative. To defend profits from sales of unhealthy foods, the food industry has adapted techniques long used by the tobacco industry to defend cigarettes. For example, they seek to instill doubt in the public about scientific evidence linking certain foods and eating patterns to obesity, emphasize personal responsibility, hire scientists to counteract obesity research, make self-regulatory pledges, lobby to stop government public health anti-obesity programs, and, of course, heavily advertise unhealthy foods [6]. At a macroscopic level in western countries, the overconsumption of value added foods (i.e., processed and refined) is a predictable outcome of market economies predicated on consumption-based growth [7].

Evidence-based public health strategies are needed to better understand the dietary and physical activity factors implicated in the development of obesity and to guide interventions and policies that can curb or reverse the increase in BMI globally [8]. Only with a quantitative understanding of the factors leading to excessive weight gain guiding the implementation of weight control education campaigns and practical public health strategies will the obesity epidemic be curbed. No methodology for relating macronutrient intake and exercise to long-term BMI change/year or future BMI has yet been scientifically validated.

A 2007 European Union regulation on nutrition and health claims made for foods provides for the use of nutrient profiles to determine which foods may bear claims but does not specify what the profiles should be or how they should be developed [9]. An in depth analysis by the French Food Safety Agency of existing nutrient profiling schemes based on indicator foods [10] found fairly good concordance between (1) The UK Food Standards Agency (FSA) model (2), The Dutch Tripartite classification model, and (3) The USA Food and Drug Administration (FDA) model but concluded, “… further improvement of the "indicator foods" approach is needed if it is to serve as a "gold standard" [9,10]. The British Heart Foundation Health Promotion Research Group compared eight nutrient profiling models with a standard ranking of 120 foods. They found good correlations with opinions of nutrition professionals about both the continuous models of nutrient profiling (Spearman's rho = 0.6-0.8) and categorical models of food profiling (high chi squared results) [11]. However, these correlations are expert opinion-based rather than evidence-based and therefore subject to challenges that they are biased.

In a consensus report entitled, “Front-of-Package Nutrition Rating Systems and Symbols: Promoting Healthier Choices,” a USA Institute of Medicine committee concluded, “…it is time for a move away from front-of-package systems that mostly provide nutrition information on foods or beverages but don’t give clear guidance about their healthfulness, and toward one that encourages healthier choices through simplicity, visual clarity, and the ability to convey meaning without written information. The report recommends that the FDA develop, test, and implement a single, standard front-of-package symbol system to appear on all food and beverage products, in place of other systems already in use” [12].

Food and Agriculture Organization (FAO) and World Health Organization (WHO) data worldwide [13,14] show large variations in diets, physical activity, and mean BMIs for female and male cohorts from countries around the world. This provides an opportunity to derive multiple regression formulas capturing the relationship between diet, physical activity, and mean BMIs of adults. Similarly, diet, exercise, and BMI data from the Diabetes Control and Complications Trial (DCCT) and the DCCT follow up study, Epidemiology Diabetes Intervention and Complications study (EDIC), can be used to generate multiple regression formulas predicting BMI change/year. By omitting the physical activity or exercise components of the formulas, the macronutrient components can be used to predict the long-term weight impacts of individual foods and beverages.

In the first and only genuinely evidence-based long-term weight impact assessment methodology of foods and beverages ever published, Harvard nutritional epidemiologists led by Dr. Dariush Mozaffarian analysed the statistical relationship between increases and decreases in servings per day of specific foods and beverages and 4 four year changes in weight of subjects from three large studies of diet and lifestyle [15]. The food group profiling component (categorical profiling) of this pioneering Harvard diet and lifestyle study serves as the comparator for results from the FAO/WHO and DCCT/EDIC macronutrient (continuous profiling) formulas for future BMI predictions for 4 four year weight impacts of individual foods and beverages.

This paper will explore whether these macronutrient and physical activity profiling multiple regression formulas are validated by comparison with the evidence-based Harvard nutritional epidemiology food group profiling study and correlated enough with each other to provide a generalisable model to predict long-term BMI change/year for diverse individuals, populations, foods, and beverages. If so, these future BMI continuous model prediction formulas could inform obesity prevention public health policies for countries and weight control strategies for clinicians and individuals.

Increased physical activity (FAO/WHO database) and exercise (DCCT/EDIC database) were hypothesized to reduce weight gain over time. Regarding the relationship between food group availability/macronutrient availability and BMI change/year of cohorts of female and male adults from countries around the world and macronutrient consumption of individuals with type 1 diabetes, an exploratory hypothesis was put forth.

## Methods

### FAO and WHO data

Of 200 countries in the Global Health Observatory Data Repository of the WHO and the FAO databases, 112 countries have complete data on plant and animal food commodity availability per capita [14], physical activity [16], and mean BMI (kg/m^2^) of adults aged 25+ in 2008 [17]. For an additional 55 countries, physical activity data was absent but diet and BMI were available. Imputed estimates of the WHO variable “insufficient physical activity” of these 55 female and male cohorts were obtained by using multiple regression analysis with insufficient physical activity of the 112 countries as the dependent variable and food group availability profile, gender, and country percapita GDP as independent variables. In all, 334 cohorts from 167 countries served as the subjects for these univariate and multivariate statistical analyses.

### FAO food group availability data and derived macronutrient profiles

The FAO supplied data on food commodity availability in kilocalories (kcals) per capita per day. These food commodity availability data were broken down to cereals (e.g., rice, maize, and corn), vegetable oils (e.g., soy, rapeseed, mustard seed, and palm), sugar and sweeteners (e.g., sucrose and fructose from sugar cane, corn, beets, and honey), meat (e.g., cow, pig, sheep, goat, offals), animal fats, roots and tubers (e.g., potatoes and cassavas), fruit (including juices), pulses (e.g., beans and lentils), milk, cheese, and eggs. The percent of total available kcals percapita per day for each food group in each country comprised that country’s food group profile. Data for percapita alcohol “consumption”, in contrast with “availability,” came as the variable “g/day consumed” from the WHO [18], rather than as percent of total available kcals.

Macronutrients included for univariate analysis with mean adult BMI were protein (g and % of kcals), carbohydrates (g and % of kcals), dietary fiber (g and g/1,000 kcals), polyunsaturated fatty acids (PUFA: g and % of kcals), monounsaturated fatty acids (MUFA: g and % of kcals), saturated fatty acids (SFA: g and % of kcals), and total fats (g and % of kcals). In addition, percapita daily total kcals was assessed for each cohort.

To generate the FAO/WHO macronutrient profiling formula, the FAO food commodity availability data were broken down to macronutrient availability by analyzing samples of each commodity using the United States Department of Agriculture (USDA) Nutrient Database for Standard Reference, Release 24 [19]. Correlations of the mean BMIs of female and male cohorts from different countries with the formula estimates served as a measure of the adequacy of the method of transforming food group data to macronutrient profile data (i.e., #1 physical activity and food group profiling versus #2 physical activity and macronutrient profiling).

### WHO physical activity data

The WHO evaluated physical activity of females and males in countries worldwide with the variable “insufficient physical activity” (0%-100% scale). According to the WHO, “adults aged 18–64 should do at least 150 minutes of moderate-intensity aerobic physical activity throughout the week or do at least 75 minutes of vigorous-intensity aerobic physical activity throughout the week or an equivalent combination of moderate- and vigorous-intensity activity” [20]. The WHO defined “insufficient physical activity” as less than this recommended level of physical activity.

### The DCCT/EDIC study

Diabetes Control and Complications Trial (DCCT) and its follow-up the Epidemiology of Diabetes Interventions and Complications (EDIC) study were conducted by the DCCT/EDIC Research Group and supported by National Institute of Health grants and contracts and by the General Clinical Research Center Program, NCRR. The data [and samples] from the DCCT/EDIC study were supplied by the NIDDK Central Repositories. This manuscript was not prepared under the auspices of the DCCT/EDIC study and does not represent analyses or conclusions of the DCCT/EDIC study group, the NIDDK Central Repositories, or the NIH.

The DCCT eligibility criteria and screening methods and the baseline characteristics of the study subjects have been reported in detail [21-23]. From 1983 and 1989, investigators recruited 1,441 participants between 13 and 39 years of age (mean = 26.8 years) that were C peptide-deficient and in good general health. The range of follow-up for all subjects was 3.5 to 9 years, with a mean of 6.5 years at the end of the trial in 1993 [24]. EDIC data from the National Institute of Diabetes and Diseases of the Kidney (NIDDK) were obtained in collaboration with the Endocrinology Department of the University of Pittsburgh. The EDIC study collected data over the 10 years immediately following the DCCT (1993–2003) [25], bringing the total length of follow up of yearly BMI records to 14–19 years.

### DCCT macronutrient profiling

Under the guidance of registered dietitians trained in collecting nutrient consumption data, all DCCT participants submitted detailed accounts of their food intake during the week previous to entry into the study. Subsequently, the dietitians obtained follow-up diet histories at years two and five and at the end of the trial [21]. With these diet histories transformed into intakes of 99 macro and micronutrients, statisticians generated a nutrient consumption data set. This nutritional analysis instrument had a high reproducibility on repeated assessments of the diet history [26]. After screening the data set for nutrients correlating with BMI change/year, univariate correlations were performed on food energy expressed as total kcals per day percapita and the same macronutrient variables as were included as in the FAO macronutrient analysis. To include as much of the macronutrient profile spectrum as possible, the variable “total fat – PUFA” was derived and included in the multiple regression analyses in place of the variables total fat, MUFA, and SFA. Otherwise only the MUFA variable would enter the formula.

For each DCCT participant, kcals and macronutrient intakes on entry, at years two and five, and on completion of the study were averaged.

### Glycemic control of DCCT participants

Since people with type 1 diabetes comprised the DCCT database, poor glycemic control could confound the relationship of diet with BMI change/year. Glycosuria due to serum blood sugar levels chronically greater than nine mmol/l reduces the BMI at the expense of increased complications of diabetes for patients. Using HbA1c <= 9.5 as the cutoff for inclusion resulted in no significant correlation between the mean HbA1c and BMI change/year (n = 1,055, r = 0.02, *P* = 0.42). However, with HbA1c > 9.5, BMI change/year decreased significantly as HbA1c rose (n = 137, r = −0.19, *P* = 0.0304). Consequently, HbA1c ≤ 9.5 was selected as the cutoff for participant inclusion in the analysis.

### Exercise in DCCT participants

In the DCCT data set, the variable “exercise” was on a four point scale (approximate scale gradation: 1 = sedentary, 2 = mild activity (i.e., brisk walking about 30 minutes/day on average or the equivalent), 3 = moderate activity (i.e., brisk walking or running about 60 minutes/day on average or the equivalent), and 4 = strenuous activity (i.e., rigorous aerobic exercise ≥ 90 minutes/day on average). Mean exercise levels while on study were the following: < 1.5: 22.2%, 1.5 - < 2.5: 70.6%, 2.5 - < 3.5: 6.0% and 3.5 - 4.0: 1.2%. For each DCCT participant, yearly exercise assessments were averaged.

### Imputing values for “insufficient physical activity” for cohorts with no data from WHO

The mean percent of insufficient physical activity varied markedly in cohorts of females and males from 112 countries (females: range = 6.6% to 76.2%, mean [90% CI] = 37.8 [10.9 to 69.7], median = 37.4 and males: range = 2.7% to 70.7%, mean [90% CI] = 31.0 [10.8 to 60.7], median = 29.3). To impute the level of adult physical activity of the 55 female and 55 male cohorts from countries without insufficient physical activity data from countries with insufficient physical activity data, a multiple regression formula was generated with insufficient physical activity as the dependent variable and the 26 food and beverage groups, mean BMI, gender, and country per capita GDP as independent variables. A constant was added to adjust the lowest imputed insufficient physical activity estimate to “2.7,” equating with the lowest of the range of reported insufficient physical activity values (Bangladeshi males). The resulting formula is as follows:

"Imputed insufficient physical activity = 1.01691 meat + 0.36409 wheat −0.14531 rice + 1.18572 Palm Oil – 2.85570 Sheep and Goats – 2.13059 Potatoes – 3.05422 Cheese + 0.00025787 Country percapita GDP in 2009 + 5.16467 Female gender + 2.65466 mean adult BMI – 38.14 (R^2^ = 0.48);"

The mean [SD] of the known values for insufficient physical activity and the imputed values were fairly similar (224 cohorts with known values for insufficient physical activity, mean [SD] = 34.4 (17.0) versus 110 cohorts with imputed values for insufficient physical activity, mean [SD] = 38.3 (11.8).

### Comparison of FAO/WHO macronutrient profiling formula results with the formula from DCCT/EDIC participants

For the purpose of comparing the FAO/WHO formula predictions of mean BMI of adults with the BMI change/year formula from the DCCT/EDIC trial, the WHO variable “insufficient physical activity” (scale: 0% – 100% of the sample) was converted to the DCCT exercise variable (1–4 scale). The formula for this conversion is as follows: Y (DCCT 1–4 exercise scale) = 1.82 (100% – Z% (WHO insufficient physical activity scale: 0% – 100%))/59.55%. “Z%”, the WHO “insufficient physical activity” score, was transformed to “Y”, the DCCT exercise value used for compatibility of the two formulas. For North American DCCT participants, “1.82” was the mean exercise level on the DCCT 1–4 scale. The divisor, “59.55%”, represents the mean amount of “sufficient physical activity” of males and females in the USA (WHO “insufficient physical activity” scores for USA males and females were 33.5% and 47.4%, respectively. The mean “insufficient physical activity” in North America was 33.5% + 47.4% = 80.9÷2 = 40.45%. Therefore, “sufficient physical activity” = 100% – 40.45% = 59.55%). The conversion of WHO activity scores to the DCCT exercise (1–4 scale) gave the following estimated means and ranges of scores for country female and male cohorts: females: mean DCCT score = 1.86, range 0.073 to 2.87 and males: mean DCCT score = 2.08, range 0.90 to 2.97. As expected due to the formula deriving DCCT “exercise” from WHO “insufficient physical activity,” these variables were negatively correlated (r= −1.0).

### Evaluation of the long-term weight impact of foods and beverages

To evaluate the long-term weight impact of individual foods and beverages, each item was assessed by adding the macronutrient profile data of one serving of that food or beverage to the mean macronutrient values of cohorts/individuals from the respective databases. To compose a weight impact prediction formula based on macronutrient profiling for individual foods and beverages comparable to the four-year weight impact (in pounds) of foods profiling analysis of Mozaffarian and his nutritional epidemiology colleagues authoring the Harvard diet and lifestyle study [15], the following adjustments were made to the FAO/WHO and DCCT/EDIC macronutrient and exercise profiling BMI change/year prediction formulas:

1. Eliminate the exercise variable

2. Switch from macronutrients as “% of kcals” to g

3. Multiply b weights of protein, carbohydrates and fats by 100 because of the change from % (0–100) to these variables expressed as a fraction of the total kcals (i.e., 0.00 - 1.00)

4. Multiply protein and total carbohydrates by “4” because they contain 4 kcals/g

5. Multiply fats by “9” because they contain 9 kcals/g

6. Multiply alcohol by “7” because it contains 7 kcals/g (DCCT/EDIC only)

7. Retain alcohol in grams as a variable in the FAO/WHO database because alcohol “consumption” data (WHO) and not “availability” data (FAO) was used

8. Multiply b weights of dietary fiber by 1,000 because of the change from dietary fiber g/1,000 kcal to dietary fiber g

9. Adjust the standard deviations (SDs) of the FAO/WHO and DCCT/EDIC formulas to the SD of the Mozaffarian predictions of the 22 food and beverage groups (SD=0.88734) by multiplying each formula by 0.88734 ÷ (the initial SD of the respective formula estimates for the 22 items).

10. Set the output of each multiple regression formula for a hypothetical food with 0.1 kcal and no macronutrients at “0.00” weight impact in four-years by adding a constant, thereby centering the weight impact of each formula at 0.00 pounds in four-years.

Assuming all macronutrient variables would enter the multiple regression macronutrient profiling formulas to predict BMI change/year (although not all macronutrient variables do enter the formulas), the formulas would have the following format:

"Weight impact over 4 years (pounds) = (b weight of kcals * kcals per serving of the food or beverage + b weight of protein * 4 (kcal/g protein) * 100 (conversion from percentages to 0.00 - 1.00 portions) * protein (g protein per serving + mean intake/day protein g)/(kcals per serving + average kcals/day) + b weight of carbohydrates * 4 (kcal/g carbohydrate) * 100 * carbohydrate (g carbohydrates per serving + mean intake/day carbohydrates g) /(kcals per serving + average kcals/day) + b weight of dietary fiber * dietary fiber (g per 1,000 kcals) * 1,000/(kcals per serving + average kcals/day) + b weight of PUFA * 9 (kcal/g PUFA) * 100 * PUFA (g PUFA per serving + mean intake/day PUFA g) /(kcals per serving + average kcals/day) + b weight of MUFA * 9 (kcal/g MUFA) * 100 * MUFA (g MUFA per serving + mean intake/day MUFA g) /(kcals per serving + average kcals/day) + b weight of SFA * 9 (kcal/g SFA) * 100 * SFA (g SFA per serving + mean intake/day SFA g) /(kcals per serving + average kcals/day) + b weight of total fat * 9 (kcal/g total fat) * 100 * total fat (g total fat per serving + mean intake/day total fat g) /(kcals per serving + average kcals/day) + b weight of alcohol * 7 (kcal/g alcohol) * 100 * alcohol (g alcohol per serving + mean intake/day alcohol g) /(kcals per serving + average kcals/day)) * 0.88734 (the SD of the weight impacts of the Mozaffarian predictions of the 22 food and beverage groups) ÷ (the initial SD of the FAO/WHO and DCCT/EDIC formula estimates, respectively, for the 22 foods and beverages) + constant;"

### Statistical analysis

Pearson correlations related food group availability (% of total kcals for each food group), availability of kcals percapita overall, and exercise (WHO insufficient physical activity data transformed to DCCT 1–4 scale) to mean BMI of females and males in each country. Similarly for the derived FAO macronutrient profiling analysis, Pearson correlations related mean BMI in each country to exercise (DCCT 1–4 scale), availability of kcals overall, protein (g and % of total kcals), carbohydrates (g and % of total kcals), dietary fiber (g and g per 1,000 kcals), PUFA (g and % of total kcals), MUFA (g and % of total kcals), SFA (g and % of total kcals), total fat (g and % of total kcals), and total fat – PUFA (g and % of total kcals). Alcohol (g/day) consumption, not availability, was obtained from the WHO. Consequently, alcohol (% of kcals) relative to the other macronutrients was not known or estimated.

Based on the Bonferonni correction for univariate correlations of food group and exercise variables related to mean BMI, *P* values less than 0.002 were considered significant (i.e., for 27 food groups and exercise: 0.05/28 = 0.00179, rounded off to 0.002).

In the multivariate analyses, WHO data on mean BMIs from female and male adults 25+ years old in 2008 from 167 countries (criterion variable) were correlated with FAO food commodity availability/macronutrient availability data and exercise (DCCT 1–4 scale) data (predictor variables).

In the DCCT analysis, BMI change/year equaled (BMI at the end of the trial – the initial BMI) ÷ years on trial. Pearson correlations were computed for food energy (kcals), macronutrients and exercise with BMI change/year. Based on the Bonferonni correction for macronutrients and exercise of both the FAO/WHO and DCCT/EDIC data sets, *P* values less than 0.002 were considered significant in univariate correlations (i.e., for 21 macronutrient and exercise variables: 0.05/21 = 0.0024).

With DCCT/EDIC data, multiple regression analysis generated a formula quantifying the relationship of BMI change/year (the criterion variable) with macronutrient intake and exercise (predictor variables).

For the FAO/WHO multiple regression analyses, predictor variables gained entry in the formulas if *P* < 0.10 and remained if *P* < 0.10. For the DCCT/EDIC formula, predictor variables gained entry in the formulas and remained if *P* < 0.25. To derive the formulas, multipliers of each significant predictor variable were the non-standardized coefficients (i.e., b weight). Constants centered each macronutrient profiling formula.

The USDA Nutrient Database for Standard Reference Release 24 [19] served as the macronutrient composition reference for the 22 categories of food and beverage groups included in the Harvard diet lifestyle study. These profiles were plugged into the two formulas to generate four-year weight impact estimates for each of these 22 foods and beverages. Pearson correlations compared Harvard diet and lifestyle study weight impact estimate data on food and beverage groups with results of the FAO/WHO four-year weight impact formula, the DCCT/EDIC four-year weight impact formula, and the average of the two formulas. Additionally, the FAO/WHO 4 four year weight impact formula and the DCCT/EDIC four-year weight impact formula were compared with each other.

SAS statistical software (release 9.1, SAS Institute, Cary, NC) was used in the performance of the data analysis.

## Results

Table 1 shows selected FAO plant and animal food group availability data (percentage of total kcals available from each major food group) of female and male cohorts from the 167 countries. Kcal intake of males was equated to 1.2 times the mean population kcal intake and for females 0.8 times the mean, in accordance with USA data from the National Health and Nutrition Examination Survey [27].

**Table 1:** **Selected FAO food availability data as percent of total available Kcals from worldwide countries**[14]

**Country**	**Kcal**	**Cereals**	**Vege Oil**	**Sugars and sweets**	**Meat and offals**	**Roots Tubers**	**Milk, Eggs, Fish**	**Fruit**	**Animal Fat**	**Pulses**	**ETOHG/day**
Afghanistan	.	.	.	.	.	.	.	.	.	.	0.04
Albania	2,904	41.99	5.89	6.95	7.78	2.13	19.67	9.15	1.99	1.55	8.75
Algeria	3,104	55.94	10.68	9.37	3.06	3.43	6.92	6.93	0.51	1.94	0.79
Andorra	.	.	.	.	.	.	.	.	.	.	12.20
Angola	1,949	36.64	10.91	8.22	5.26	26.57	2.48	2.71	0.45	2.86	6.68
Antigua and Barbuda	2,319	28.02	10.17	13.43	14.70	1.41	14.72	9.14	2.56	1.11	9.80
Argentina	3,001	34.79	9.52	15.69	17.05	3.22	8.56	4.54	2.38	0.33	11.22
Armenia	2,250	52.07	3.93	8.09	6.71	6.40	8.41	8.74	3.03	0.00	16.39
Australia	3,186	22.45	14.87	13.65	16.65	2.90	11.33	6.26	4.61	0.17	12.25
Austria	3,760	25.05	12.12	12.43	13.30	3.11	8.90	6.48	8.42	0.16	14.88
Azerbaijan	2,996	54.93	4.79	7.81	4.59	5.96	8.96	6.87	1.64	0.00	16.01
Bahamas	2,701	27.68	5.88	15.37	18.05	1.92	7.92	9.02	5.64	0.52	10.38
Bahrain	.	.	.	.	.	.	.	.	.	.	5.03
Bangladesh	2,250	79.90	6.40	3.75	0.67	2.15	2.37	1.25	0.27	1.84	0.20
Barbados	3,055	30.82	8.24	17.46	11.65	3.11	9.71	5.83	1.82	2.21	7.70
Belarus	3,086	33.05	8.02	10.56	10.78	11.43	11.36	4.72	5.10	0.00	22.62
Belgium	3,690	22.05	14.62	13.95	7.90	3.89	12.27	5.68	11.12	0.54	12.49
Belize	2,719	35.56	3.18	15.83	8.16	1.96	6.76	13.10	4.35	3.76	7.10
Benin	2,512	39.03	8.12	1.88	2.22	32.30	1.61	2.69	0.62	3.78	2.50
Bermuda	.	.	.	.	.	.	.	.	.	.	.
Bhutan	.	.	.	.	.	.	.	.	.	.	0.65
Bolivia	2,093	46.00	3.20	13.00	10.90	6.70	3.30	7.20	2.80	1.20	6.94
Bosnia and Herzegovina	3,084	48.05	6.14	4.78	3.95	5.38	9.55	7.75	1.14	2.05	11.52
Botswana	2,235	45.14	8.88	12.68	4.42	7.06	6.98	1.78	1.54	6.18	8.36
Brazil	3,099	32.85	12.88	13.17	12.34	4.33	7.48	4.73	1.84	4.84	12.10
Brunei Darussalam	2,987	45.16	8.57	11.55	8.46	1.47	10.45	5.18	1.28	0.71	2.23
Bulgaria	2,761	37.20	15.92	11.11	7.70	2.36	10.79	3.99	2.51	1.13	13.68
Burkina Faso	2,669	72.53	4.58	1.74	3.40	0.68	1.50	0.69	0.57	4.12	8.78
Burundi	1,680	17.28	0.97	1.63	1.23	36.12	0.67	15.76	0.25	18.85	11.58
Cambodia	2,245	73.16	2.34	3.89	5.75	3.48	2.90	2.23	0.83	0.94	5.65
Cameroon	2,259	39.25	9.29	4.39	3.08	17.46	2.48	9.47	0.28	5.96	9.48
Canada	3,530	24.27	15.79	14.75	10.62	3.92	8.27	5.79	7.21	1.98	12.24
Cape Verde	2,549	47.76	8.49	9.66	8.28	3.80	8.64	3.69	2.33	2.64	5.98
Central African Republic	1,956	23.12	14.04	4.58	8.45	30.97	1.96	4.16	1.09	3.37	3.80
Chad	2,040	53.15	5.38	3.98	3.17	8.43	2.79	1.21	0.47	4.48	5.27
Chile	2,957	38.62	10.96	14.62	12.73	3.46	7.61	5.14	2.30	1.43	10.57
China	2,974	50.79	6.10	2.25	14.66	6.35	4.78	7.81	1.36	0.38	6.67
Colombia	2,662	33.56	10.34	18.64	6.73	6.07	9.11	9.14	1.20	2.07	7.91
Comoros	1,857	37.12	11.07	5.98	2.53	15.47	3.86	8.02	0.20	8.64	0.34
Congo	2,513	26.55	14.36	6.16	3.69	33.38	3.29	6.19	0.13	1.43	5.35
Congo, Dem Rep	1,585	20.24	7.45	1.88	1.25	56.24	0.86	3.73	0.11	2.09	4.07
Costa Rica	2,813	33.71	11.48	19.84	4.99	1.88	11.62	5.51	2.74	3.47	6.97
Cote d'Ivoire	2,515	30.55	12.51	4.48	2.18	32.61	2.04	8.39	0.13	0.70	7.76
Croatia	2,987	30.41	12.45	15.61	6.21	5.08	11.44	5.49	2.85	0.71	18.00
Cuba	3,295	40.72	5.37	16.73	4.68	7.60	4.77	9.01	0.65	6.80	6.14
Cyprus	3,199	23.27	12.03	14.24	13.35	2.53	13.58	7.47	1.45	1.34	10.61
Czech Republic	3,317	28.66	12.57	13.26	10.31	4.12	9.77	4.27	4.90	0.69	19.76
Denmark	3,397	23.95	4.34	14.28	13.11	3.99	11.74	6.09	12.65	0.29	14.42
Djibouti	.	.	.	.	.	.	.	.	.	.	2.24
Dominica	3,115	24.56	5.35	13.90	11.25	9.00	10.79	11.54	1.22	1.67	10.42
Dominican Republic	2,263	28.86	16.56	16.08	7.04	2.55	7.13	10.01	1.47	3.61	7.54
Ecuador	2,304	33.33	15.19	8.00	9.68	3.20	8.32	14.03	3.76	1.84	11.32
Egypt	3,163	63.70	4.20	8.25	2.86	1.70	3.56	8.44	1.51	2.53	0.38
El Salvador	2,585	49.55	4.96	15.09	3.51	2.03	7.83	5.27	2.54	4.84	4.79
Equatorial guinea	.	.	.	.	.	.	.	.	.	.	7.34
Eritrea	1,587	67.58	10.12	3.25	3.07	3.76	1.73	0.42	0.54	7.54	1.97
Estonia	3,129	26.81	6.22	16.38	8.69	6.18	14.46	4.85	4.20	0.48	20.69
Ethiopia	1,952	66.15	1.92	2.40	2.62	13.62	2.04	1.45	0.81	6.70	4.92
Faeroe Islands	.	.	.	.	.	.	.	.	.	.	.
Fiji	3,033	40.64	8.80	11.37	8.17	7.87	5.24	2.33	4.09	2.87	3.31
Finland	3,215	27.65	7.99	10.40	16.00	4.25	16.45	4.49	4.20	0.36	15.72
France	3,553	25.18	12.53	10.52	14.19	3.25	14.02	5.50	7.35	0.50	14.98
French Polynesia	2,904	30.28	11.21	7.20	16.41	3.87	10.58	3.79	3.95	0.98	.
Gabon	2,730	33.24	5.85	5.46	8.01	17.71	5.05	13.83	0.63	0.27	11.35
Gambia	2,345	52.53	20.41	11.14	1.51	1.08	4.41	1.10	0.18	1.41	4.30
Georgia	2,813	56.11	4.41	6.56	6.69	4.13	11.50	4.76	2.10	0.00	7.99
Germany	3,530	24.92	11.52	13.95	10.21	4.03	11.41	5.04	9.04	0.18	14.57
Ghana	2,849	29.85	6.35	3.22	1.43	39.61	2.57	10.75	0.21	0.19	3.73
Greece	3,700	28.87	16.69	9.10	8.61	3.70	12.66	10.01	1.14	1.18	13.21
Greenland	.	.	.	.	.	.	.	.	.	.	.
Grenada	.	.	.	.	.	.	.	.	.	.	12.85
Guatemala	2,171	52.13	8.10	17.56	3.96	0.82	4.13	4.68	1.03	3.96	8.52
Guinea	2,529	47.45	14.26	4.67	1.45	14.08	1.97	10.70	0.19	2.25	0.95
Guinea-Bissau	2,288	60.13	12.51	4.01	4.50	9.13	1.43	4.03	0.50	0.61	4.68
Guyana	2,753	45.56	5.50	12.55	6.09	4.17	9.68	5.29	0.24	2.19	10.44
Haiti	1,848	49.45	5.59	11.30	4.38	8.05	2.30	6.96	0.89	4.73	7.10
Honduras	2,601	45.55	9.47	16.72	5.74	0.59	7.29	6.50	1.92	3.91	5.32
Hong Kong	.	.	.	.	.	.	.	.	.	.	.
Hungary	3,438	25.61	12.43	13.03	10.24	3.53	8.66	5.84	11.55	1.09	19.34
Iceland	3,330	19.05	5.64	14.00	16.45	2.89	23.00	4.83	5.42	0.30	8.86
India	2,301	58.22	10.39	9.84	0.94	1.76	4.71	3.64	2.41	4.56	3.23
Indonesia	2,535	63.92	6.79	6.37	2.41	6.09	2.88	4.23	0.35	0.52	0.67
Iran	3,042	55.85	6.12	8.50	4.60	3.66	4.72	10.79	1.92	2.15	1.24
Iraq	.	.	.	.	.	.	.	.	.	.	0.56
Ireland	3,532	25.80	10.13	12.18	12.80	5.47	13.91	4.55	4.63	0.67	17.90
Isle of Man	.	.	.	.	.	.	.	.	.	.	.
Israel	3,540	33.09	18.77	8.02	11.80	2.44	7.71	9.17	1.43	1.73	3.02
Italy	3,657	31.21	17.84	8.21	11.03	1.98	10.36	7.79	4.41	1.41	11.66
Jamaica	2,848	31.70	10.65	17.65	8.83	5.57	8.62	7.97	2.32	0.96	6.20
Japan	2,806	38.55	12.17	10.19	6.42	2.43	12.60	4.65	1.35	0.62	9.35
Jordan	2,977	44.87	16.15	14.97	5.04	1.74	6.26	5.10	0.36	2.68	0.78
Kazakhstan	3,359	43.21	8.30	10.25	9.27	6.10	14.03	3.74	1.75	0.11	13.32
Kenya	2,060	50.19	7.77	9.08	4.09	6.08	7.40	5.91	0.38	6.71	4.66
Kiribati	.	.	.	.	.	.	.	.	.	.	3.24
Korea, Dem. Rep.	2,146	61.03	5.61	1.99	4.71	6.88	1.89	7.43	0.50	5.43	5.21
Korea, Rep.	3,074	44.33	11.56	11.08	7.28	1.15	5.54	8.02	1.94	0.48	17.77
Kosovo	.	.	.	.	.	.	.	.	.	.	.
Kuwait	3,038	40.48	16.21	11.90	11.03	1.07	6.37	5.26	1.87	2.10	0.12
Kyrgyz Republic	2,672	56.27	3.08	5.88	6.72	8.38	11.37	4.40	0.30	1.47	5.66
Laos	2,227	72.24	1.45	1.99	5.01	3.14	2.35	5.57	0.63	1.17	8.39
Latvia	3,019	28.73	11.26	10.77	8.50	7.13	14.54	4.66	6.76	0.00	16.14
Lebanon	3,107	33.65	13.69	10.50	8.06	5.96	6.60	9.23	1.88	2.62	2.76
Lesotho	2,468	79.45	1.79	6.21	3.49	3.38	1.36	1.00	0.40	1.70	6.67
Liberia	2,163	39.98	19.98	2.43	2.08	24.16	0.91	4.93	0.11	1.34	6.08
Libya	3,144	43.36	17.15	11.92	4.15	2.15	7.60	7.50	0.27	1.35	0.12
Liechtenstein	.	.	.	.	.	.	.	.	.	.	.
Lithuania	3,419	35.12	8.26	9.91	9.78	6.51	12.91	4.60	4.51	0.79	19.56
Luxembourg	3,685	21.52	9.09	7.39	21.33	2.16	15.45	6.14	0.99	0.23	15.41
Macao SAR, China	.	.	.	.	.	.	.	.	.	.	.
Macedonia, FYR	2,983	36.03	13.30	12.03	7.39	3.42	5.72	7.79	5.63	1.91	10.73
Madagascar	2,133	58.28	3.91	3.18	3.78	20.38	3.01	3.47	0.44	1.95	1.58
Malawi	2,127	55.86	2.65	5.58	1.52	18.04	0.94	5.85	0.17	6.10	1.73
Malaysia	2,908	45.43	13.13	12.12	8.85	2.07	8.69	3.70	0.80	0.92	1.04
Maldives	.	.	.	.	.	.	.	.	.	.	.
Mali	2,579	67.43	7.63	4.71	4.19	1.91	5.38	2.11	0.44	4.46	1.19
Malta	3,592	33.40	4.88	15.21	9.16	3.22	12.00	7.44	5.52	1.47	4.92
Marshall Islands	.	.	.	.	.	.	.	.	.	.	.
Mauritania	2,823	46.77	11.68	15.45	5.58	0.52	11.94	1.71	1.09	4.50	0.13
Mauritius	2,936	47.39	13.62	11.84	6.01	1.19	7.74	3.47	0.87	3.29	4.24
Mexico	3,245	43.93	7.82	15.41	9.36	0.98	7.73	4.95	2.24	3.86	10.26
Micronesia	.	.	.	.	.	.	.	.	.	.	6.30
Moldova	2,907	48.25	5.01	9.31	4.95	4.70	11.25	5.26	2.04	0.09	27.61
Monaco	.	.	.	.	.	.	.	.	.	.	.
Mongolia	2,254	45.41	6.25	7.07	18.72	3.45	10.73	1.52	3.21	0.16	4.09
Montenegro	2,445	.	.	.	.	.	.	.	.	.	.
Morocco	3,230	61.58	7.97	11.22	3.02	2.45	2.65	5.96	1.39	1.93	1.49
Mozambique	2,071	44.73	8.67	3.21	1.40	34.18	0.65	1.11	0.13	3.91	2.72
Myanmar	2,438	60.51	9.08	5.60	4.84	1.11	3.58	4.47	0.68	5.61	0.70
Namibia	2,349	45.17	5.63	10.32	6.75	13.88	6.15	1.40	2.29	2.92	13.75
Nepal	2,349	68.20	8.35	1.80	1.72	4.05	3.66	3.60	1.23	3.15	2.90
Netherlands	3,243	19.09	13.30	14.00	10.49	4.91	17.43	7.71	4.77	0.48	11.70
New Caledonia	2,789	32.18	13.04	8.28	12.32	5.16	10.28	3.80	2.88	0.69	.
New Zealand	3,150	23.73	6.61	18.43	14.78	3.72	9.38	7.47	8.35	1.38	11.99
Nicaragua	2,400	53.51	8.56	15.52	3.95	1.24	6.06	1.65	0.66	6.35	6.25
Niger	2,306	65.96	5.56	3.37	2.40	1.81	2.32	2.46	0.90	8.79	0.41
Nigeria	2,708	44.44	13.21	3.81	1.45	19.34	1.47	4.72	0.23	3.10	15.26
Norway	3,455	28.12	11.57	12.32	11.20	4.03	13.81	5.13	6.59	0.39	10.02
Oman	.	.	.	.	.	.	.	.	.	.	1.10
Pakistan	2,251	48.91	11.16	11.58	2.59	1.14	12.07	3.02	5.03	2.67	0.06
Palau	.	.	.	.	.	.	.	.	.	.	11.83
Panama	2,451	43.34	8.55	12.32	8.24	2.37	9.00	5.64	3.21	1.98	8.76
Papua New Guinea	.	.	.	.	.	.	.	.	.	.	4.37
Paraguay	2,622	28.97	13.90	9.46	8.80	13.38	7.02	3.71	2.97	4.43	9.49
Peru	2,426	44.32	5.25	8.84	5.08	14.35	5.73	7.32	0.83	2.72	7.84
Philippines	2,518	54.80	4.11	10.79	8.91	3.07	4.52	7.58	1.85	0.57	7.30
Poland	3,397	34.98	8.56	12.81	11.23	7.08	8.92	4.13	6.43	0.47	17.32
Portugal	3,583	28.72	11.49	8.32	11.07	4.21	11.47	7.11	6.67	1.00	16.67
Qatar	.	.	.	.	.	.	.	.	.	.	1.55
Romania	3,510	40.07	9.95	7.62	7.72	5.27	14.41	6.55	2.05	0.63	19.38
Russian Federation	3,272	37.70	8.50	12.32	7.83	7.55	11.41	3.91	3.30	0.52	19.48
Rwanda	2,054	16.37	3.55	0.84	1.43	39.30	1.50	18.47	0.28	11.79	11.99
Samoa	2,878	19.73	5.52	9.39	16.99	9.56	6.01	8.04	4.66	0.01	5.41
San Marino	.	.	.	.	.	.	.	.	.	.	.
Sao Tome and Principe	2,662	33.79	7.93	7.31	2.36	15.20	4.07	11.74	0.09	2.24	10.14
Saudi Arabia	3,133	48.28	12.11	10.24	7.25	1.09	6.10	10.03	1.14	1.25	0.41
Senegal	2,318	61.89	14.47	4.89	2.79	2.99	4.86	2.49	0.60	0.83	0.61
Serbia and Montenegro	2,703	23.4	10.8	10.2	16.3	3.0	10.7	8.0	7.8	2.7	8.59
Seychelles	2,426	41.40	8.20	10.30	6.10	1.60	11.00	6.40	2.40	2.20	14.53
Sierra Leone	2,128	49.80	14.70	2.80	1.30	9.90	3.00	3.30	0.10	5.30	11.38
Singapore	.	.	.	.	.	.	.	.	.	.	1.85
Slovakia	2,885	33.04	12.17	12.45	9.10	4.36	6.83	4.15	8.78	0.57	15.97
Slovenia	3,220	32.69	11.87	6.13	12.23	3.26	10.94	7.89	6.45	0.63	17.93
Solomon Islands	2,434	35.74	2.82	2.76	2.69	35.83	3.42	2.61	0.74	3.13	1.64
Somalia	1,762	41.36	3.97	14.27	8.44	1.55	22.35	2.30	2.55	1.09	0.60
South Africa	2,986	52.53	11.14	10.90	8.50	1.95	4.14	2.73	0.51	1.05	12.19
Spain	3,271	21.46	20.24	9.71	13.70	3.96	11.86	6.80	2.16	1.39	14.27
Sri Lanka	2,392	56.04	2.21	12.33	1.19	2.24	4.49	3.81	0.37	2.78	0.97
St. Kitts and Nevis	2,452	25.71	9.16	17.17	15.80	2.29	11.21	4.73	1.48	2.22	12.74
St. Lucia	2,744	30.16	4.19	12.41	16.70	2.97	10.86	7.89	2.91	1.91	14.46
St. Vincent and the Grenadines	2,806	34.72	9.25	16.04	12.30	4.88	5.98	6.85	0.51	1.83	5.99
Sudan	2,266	48.75	5.18	9.53	5.51	1.18	18.33	3.65	0.86	3.24	1.43
Suriname	2,468	41.40	13.72	17.90	7.09	2.11	4.33	5.67	0.73	0.63	7.87
Swaziland	2,307	46.27	4.50	13.49	7.93	5.15	6.94	3.71	1.00	2.80	6.06
Sweden	3,116	25.09	11.19	13.34	10.85	3.23	17.61	5.46	6.31	0.48	11.98
Switzerland	3,421	21.80	12.49	16.88	14.01	2.37	13.01	4.72	5.95	0.41	13.69
Syrian Arab Rep.	3,049	45.42	13.36	11.68	4.46	1.68	7.77	5.70	2.30	2.53	1.79
Tajikistan	2,127	63.00	11.30	7.20	3.80	2.80	5.20	4.70	0.10	0.50	4.07
Tanzania	2,017	53.21	6.68	4.40	2.60	17.39	3.03	3.35	0.48	4.77	9.43
Thailand	2,529	48.03	6.32	13.12	6.61	1.83	5.27	5.81	0.55	1.17	8.50
Timor-Leste	2,016	65.50	1.52	1.94	8.59	12.41	3.13	1.64	0.49	1.85	0.89
Togo	2,146	48.55	9.55	2.62	1.64	25.63	1.27	1.21	0.20	3.57	2.30
Tonga	.	.	.	.	.	.	.	.	.	.	4.70
Trinidad and Tobago	2,713	35.76	10.55	20.76	6.47	2.35	7.94	4.22	2.96	3.34	7.39
Tunisia	3,312	49.46	15.35	10.14	3.47	1.82	6.31	7.01	0.68	2.54	1.26
Turkey	3,482	49.05	13.72	7.59	2.66	3.05	7.35	8.17	1.36	3.19	3.62
Turkmenistan	2,754	.	.	.	.	.	.	.	.	.	6.00
Tuvalu	.	.	.	.	.	.	.	.	.	.	2.57
Uganda	2,247	21.28	6.22	3.44	3.07	22.20	2.65	17.84	0.38	8.12	19.37
Ukraine	3,230	38.93	9.40	13.61	5.92	8.17	10.68	4.06	3.39	0.74	20.96
United Arab Emirates	3,138	43.41	6.60	11.97	10.79	0.74	8.57	7.96	1.40	3.58	0.62
United Kingdom	3,442	25.31	11.80	11.25	13.73	6.17	12.42	5.58	4.32	0.99	15.89
USA	3,770	21.71	17.84	17.06	12.07	2.75	12.36	5.07	2.86	1.10	11.64
Uruguay	2,818	41.53	7.09	12.18	13.51	3.75	9.70	4.35	2.16	0.98	10.79
Uzbekistan	2,525	58.17	11.45	2.29	6.32	2.34	11.42	5.42	0.77	0.01	4.33
Vanuatu	2,722	32.10	4.67	5.40	8.44	18.60	3.93	6.61	1.46	0.00	1.15
Venezuela	2,582	37.59	15.44	15.46	8.36	2.68	6.28	5.61	1.32	1.67	9.12
Viet Nam	2,769	67.55	2.37	4.57	10.53	1.39	2.55	4.74	1.15	0.93	4.69
Yemen	2,032	58.79	10.48	13.24	4.19	0.94	3.67	3.77	0.86	2.37	0.24
Zambia	1,885	61.91	6.21	6.28	3.24	14.16	1.97	1.47	0.38	1.35	4.27
Zimbabwe	2,207	55.81	10.62	12.59	3.85	2.26	1.65	1.04	2.00	2.34	5.95

Table 2 displays the percent of “insufficient physical activity” (WHO female and male cohort data from 2009: mean [95% CI of the mean] on a 0% to 100% scale), the mean [95% CI of the mean] BMI of female and male cohorts, and the percapita GDP of the 167 countries.

**Table 2:** **“Insufficient physical activity” and mean BMI from countries and per capita gross domestic product**[17]

**Country**	Insufficient activity males [95% CI of the mean] *= imputed	Insufficient activity females [95% CI of the mean] *= imputed	**Mean BMI for adult males 2008**	**95% CI of the mean**	**Mean BMI for adult males 2008**	**95% CI of the mean**	**Per-capita GDP 2009 $**
Afghanistan			20.7	18.3 – 23.1	21	18.0 – 24.1	425
Albania	41.5*	44.0*	26.6	25.0 – 28.3	25.6	23.6 – 27.7	3,773
Algeria	31.9 [29.5, 34.3]	49.2 [47.2-51.2]	24.6	23.6 – 25.7	26.4	25.0 – 27.9	4,022
Andorra			27.6	25.4 – 29.8	26.4	23.5 – 29.2	44,952
Angola	30.5*	39.2*	22.2	19.6 – 24.8	23.5	20.3 – 26.6	4,069
Antigua and Barbuda	49.0*	59.0*	26	23.6 – 28.3	27.8	24.7 – 30.8	14,273
Argentina	65.8 [31.8-86.6]	70.9 [36.3-89.0]	27.5	26.3 – 28.7	27.5	26.0 – 29.0	7,665
Armenia	35.3*	47.1*	25.4	24.5 – 26.2	27.3	26.2 – 28.4	2,803
Australia	35.9 [14.9-68.5]	39.9 [16.6-72.2]	27.6	27.0 – 28.1	26.9	26.2 – 27.6	42,131
Austria	30.3 [11.7-63.8]	39.2 [15.9-72.5]	26.4	24.7 – 28.2	25.1	22.7 – 27.5	45,638
Azerbaijan	31.4*	42.1*	25.6	24.9 – 26.4	27.7	26.6 – 28.7	4,950
Bahamas	51.2*	61.7*	27.4	25.1 – 29.7	29.4	26.4 – 32.4	20,916
Bahrain			27.8	26.6 – 29.0	28.7	27.2 – 30.3	17,609
Bangladesh	2.7 [2.3-3.3]	6.6 [5.9-7.3]	20.5	19.1 – 21.9	20.5	19.8 – 21.3	608
Barbados	38.3 [34.4-42.0]	55.6 [51.2-60.0]	26.6	25.0 – 28.2	29.7	27.5 – 31.9	13,181
Belarus	24.7*	31.2*	26.2	23.8 – 28.7	26.7	23.5 – 29.8	5,183
Belgium	40.4 [17.4-72.4]	45.0 [21.4-76.9]	26.8	25.6 – 27.9	25.2	23.6 – 26.8	43,799
Belize	43.4*	56.0*	27.1	26.2 – 28.0	29.9	28.8 – 31.1	4,049
Benin	7.1 [6.3-8.0]	11.2 [10.2-12.3]	22.4	21.8 – 23.0	23.7	23.0 – 24.5	772
Bermuda			.	.	.	.	88,747
Bhutan			22.8	21.4-24.3	22.9	21.1-24.6	1,772
Bolivia	31.0*	42.6*	24.5	23.0-26.1	26.9	26.0-27.7	1,774
Bosnia and Herzegovina	30.3 [10.8-60.3]	37.0 [15.1-70.4]	26.8	25.7-27.8	26.4	25.0-27.7	4,525
Botswana	26.3 [24.2-28.4]	44.1 [41.3-46.9]	22.1	21.4-22.9	26.1	25.1-27.0	5,790
Brazil	47.2 [20.4-77.1]	51.6 [22.6-79.9]	25.8	25.2-26.4	26	25.3-26.7	8,251
Brunei Darussalam	45.3*	47.0*	24.2	21.7-26.6	22.9	19.7-26.2	27,390
Bulgaria	24.7 [9.0-58.5]	28.8 [11.1-62.4]	26.6	25.7-27.6	25.5	24.2-26.8	6,403
Burkina Faso	14.6 [4.6-37.9]	16.3 [5.4-40.5]	21.2	19.7-22.8	21.6	20.6-22.7	509
Burundi	21.9*	26.0*	21.7	19.2-24.2	21.3	18.3-24.3	6,403
Cambodia	11.4 [10.4-12.5]	11.1 [9.7-12.5]	21.3	20.6-21.9	21.6	20.9-22.2	744
Cameroon	33.0 [9.2-54.4]	48.3 [18.7-73.8]	23.7	22.6-24.8	24.9	24.0-25.9	1,157
Canada	32.3 [17.7-65.9]	35.4 [14.2-69.2]	27.5	27.0-28.0	26.7	26.0-27.4	39,644
Cape Verde	12.1 [9.4-14.8]	29.4 [26.9-31.1]	23.5	22.8-24.2	25	24.0-26.0	3,228
Central African Republic	26.6*	35.2*	21.1	18.6-23.7	22.4	20.3-24.5	459
Chad	22.8 [8.1-54.6]	26.2 [9.8-59.7]	21.5	20.1-22.9	22	20.9-23.0	625
Chile	48.1*	55.9*	27	26.1-28.0	28	26.8-29.1	9,487
China	29.7 [28.6-30.8]	32.3 [31.2-33.3]	22.9	22.5-23.4	22.9	22.3-23.6	3,749
Colombia	39.7*	48.0*	25	24.4-25.6	26.3	25.6-27.0	5,166
Comoros	6.1 [1.5-17.6]	10.6 [3.4-30.0]	22.2	19.9-24.4	22.4	20.7-24.2	748
Congo	44.4 [17.5-74.5]	52.9 [24.8-81.5]	21.9	20.3-23.5	23.2	21.9-24.4	2,431
Congo, Dem Rep	44.4*	52.9*	21.8	20.3-23.3	21.7	20.8-22.6	175
Costa Rica	41.5*	48.3*	26.5	25.6-27.5	27.1	25.9-28.2	6,373
Cote d'Ivoire	28.8 [12.0, 63.8]	36.9 [15.6-71.8]	22.6	21.2-24.0	23.8	22.5-25.1	1,191
Croatia	26.2 [9.9-59.7]	21.0 [8.1-50.2]	26.8	25.4-28.2	25.2	23.3-27.1	14,323
Cuba	32.4*	42.1*	25.2	24.0-26.4	26.9	25.3-28.5	5,565
Cyprus	48.1 [20.9-78.0]	62.6 [29.5-85.6]	27.5	26.3-28.6	25.9	24.3-27.5	29,428
Czech Republic	27.6 [10.8-56.2]	22.3 [8.2-49.3]	28	27.2-28.8	26.6	25.6-27.6	18,137
Denmark	34.8 [13.6-68.5]	35.4 [14.1-69.3]	26.1	24.9-27.3	25.1	23.4-26.9	55,933
Djibouti			23.4	21.0-25.7	24.3	21.3-27.4	1,203
Dominica	14.3 [10.5-18.4]	34.4 [31.3-37.9]	24.6	23.9-25.4	28.9	27.8-29.9	6,861
Dominican Republic	57.0 [26.7-83.2]	62.9 [30.0-85.5]	25.4	24.1-26.8	27.3	25.7-28.9	4,776
Ecuador	37.0 [14.7-70.9]	48.3 [20.3-78.1]	25.6	24.0-27.3	27.1	25.8-28.4	3,648
Egypt	39.1*	53.1*	26.8	25.8-27.7	30.1	29.5-30.7	2,371
El Salvador	34.3*	43.5*	26.4	24.9-27.9	27.9	27.0-28.8	3,354
Equatorial guinea	.	.	23.7	21.0-26.5	24.5	21.1-27.9	17,944
Eritrea	26.0 [23.0-29.2]	54.8 [51.4-58.0]	20.9	20.0-21.8	21.1	20.0-22.2	364
Estonia	15.7 [6.1-44.4]	18.8 [7.0-49.3]	26.3	24.9-27.7	25.3	23.4-27.1	14,375
Ethiopia	16.5 [5.2-43.0]	22.1 [7.5-52.4]	20.3	19.2-21.4	20.7	19.9-21.6	394
Faeroe Islands	.	.	.	.	.	.	45,206
Fiji	37.3*	50.2*	26.5	25.4-27.6	29.4	27.9-30.9	3,377
Finland	40.8 [16.9-73.1]	34.9 [14.0-68.3]	26.8	26.0-27.5	25.6	24.7-26.6	45,085
France	27.7 [25.5-30.1]	37.2 [35.0-39.5]	25.9	25.2-26.5	24.8	24.0-25.7	40,663
French Polynesia	.	.	.	.	.	.	.
Gabon	26.8 [6.1-42.3]	46.4 [15.0-68.2]	24.1	23.2-25.1	26	25.0-27.0	7,411
Gambia	20.4 [18.5-22.3]	28.7 [26.8-30.6]	21.7	20.3-23.0	24.8	23.1-26.6	436
Georgia	21.1 [19.3-23.0]	23.5 [22.3-24.7]	25.6	23.2-27.9	26.5	23.4-29.6	2,441
Germany	27.5 [10.3-60.6]	28.5 [10.7-62.3]	27.2	26.4-28.0	25.7	24.7-26.7	40,275
Ghana	14.4 [13.0-15.8]	20.8 [19.1-22.5]	22.9	22.3-23.4	24.3	23.8-24.9	1,098
Greece	16.7 [5.4-37.8]	14.5 [4.7-41.4]	26.4	25.3-27.5	25	23.5-26.4	28,521
Greenland			.	.	.	.	22,508
Grenada			25.4	23.1-27.7	27.6	24.7-30.6	7,311
Guatemala	15.3 [5.0-42.9]	17.0 [5.7-46.4]	25.3	24.1-26.5	26.8	25.6-28.0	2,685
Guinea	6.1 [1.9-18.6]	18.1 [5.6-37.5]	22.5	20.2-24.7	22.5	21.4-23.5	427
Guinea-Bissau	23.9*	32.0*	21.6	19.4-23.9	22.9	19.9-25.9	562
Guyana	32.1*	45.0*	23.9	21.4-26.2	26.8	23.7-29.9	2,690
Haiti	30.8*	34.9 *	23.9	21.5-26.2	23.5	22.4-24.5	657
Honduras	45.0*	44.4*	25.1	23.6-26.7	26.7	25.8-27.7	1,903
Hong Kong			.	.	.	.	29,882
Hungary	26.4 [10.7-60.5]	25.6 [10.4-58.7]	27.3	25.8-28.9	25.9	23.6-28.2	12,635
Iceland	23.5*	25.5*	27.2	25.8-28.8	26	23.9-28.1	38,033
India	12.7 11.8-13.7]	18.4 [17.5-19.3]	21	20.5-21.5	21.3	20.8-21.9	1,192
Indonesia	31.5 [28.4-34.8]	28.1 [25.4-30.9]	21.9	21.2-22.6	23	22.1-23.9	2,272
Iran	27.1 [24.4-27.8]	47.0 [46.2-47.8]	25.3	24.8-25.8	27.2	26.6-27.9	4,526
Iraq	62.8 [60.6-64.9]	54.0 [52.0-55.9]	26.7	25.9-27.5	28.4	27.2-29.6	2,097
Ireland	47.8 [20.7-78.3]	58.5 [27.0-83.4]	27.7	27.1-28.2	26.6	25.8-27.4	49,738
Isle of Man			.	.	.	.	50,191
Israel	50.5*	56.2*	27.1	26.4-27.9	27.3	26.3-28.2	26,102
Italy	49.6 [21.9-79.6]	59.8 [28.3-84.8]	26.5	25.8-27.2	24.8	24.0-25.7	35,073
Jamaica	43.6 [18.5-75.2]	51.5 [23.2-80.1]	24.5	23.9-25.2	28.7	27.8-29.6	4,665
Japan	58.9 [24.6-81.1]	61.6 [26.3-83.7]	23.5	23.1-23.9	21.9	21.3-22.4	39,456
Jordan	47.2*	56.9*	27.5	26.8-28.2	29.2	28.7-29.8	4,242
Kazakhstan	32.0 [12.8-66.3]	31.0 [12.7-65.7]	26.3	24.3-28.3	26.8	25.0-28.6	7,241
Kenya	15.1 [4.6-40.4]	18.0 [6.1-46.8]	21.6	19.7-23.6	23	22.2-23.8	744
Kiribati	38.4 [34.8-42.1]	54.9 [51.7-58.2]	29.2	28.2-30.2	31.3	30.0-32.7	1,306
Korea, Dem. Republic	13.0*	16.1*	22.2	19.6-24.8	21.3	17.8-24.7	200
Korea, Rep.	38.0*	41.5*	24.0	23.5-24.5	23.3	22.6-24.1	17,110
Kosovo			.	.	.	.	3,011
Kuwait	56.9 [54.4-59.2]	72.1 [68.8-75.3]	29.1	28.4-29.9	31.1	30.2-32.1	41,365
Kyrgyz Republic	28.2*	36.0*	24.8	23.1-26.5	25.8	23.9-27.6	881
Laos	16.7 [5.3-43.5]	21.0 [7.3-51.0]	21.1	19.8-22.5	22	20.9-23.2	966
Latvia	28.1 [10.9-60.9]	35.9 [14.0-69.7]	26.5	25.1-28.0	25.6	23.7-27.6	11,476
Lebanon	51.9 [48.5-55.2]	41.7 [38.8-44.7]	27.4	26.8-28.0	27.2	26.4-28.0	8,321
Lesotho	20.5*	38.6*	21.9	19.5-24.3	26.8	25.6-28.0	800
Liberia	39.8*	48.4*	21.9	19.6-24.2	23.2	22.2-24.3	229
Libya	37.3 [34.9-39.7]	54.4 [52.1-56.7]	26.5	25.9-27.2	29.2	28.3-30.1	9,957
Liechtenstein			.	.	.	.	134,916
Lithuania	20.3 [16.9-24.4]	24.9 [21.4-28.8]	26.9	25.5-28.4	26.1	24.1-28.1	11,034
Luxembourg	49.9 [21.3-81.2]	45.5 [18.4-77.6]	27.4	25.6-29.2	26.1	23.6-28.6	104,354
Macao SAR, China			.	.	.	.	40,105
Macedonia, FYR	35.6*	40.8*	26.6	24.3-28.9	25.4	23.3-27.4	4,528
Madagascar	18.3 [16.6-20.1]	28.3 [26.4-30.3]	21.5	20.5-22.4	20.7	20.1-21.4	422
Malawi	7.3 [6.0-8.6]	13.2 [11.9-14.6]	22	21.4-22.7	22.9	22.3-23.6	327
Malaysia	57.3 [54.3-60.4]	65.6 [63.1-67.9]	24.7	24.1-25.3	25.4	24.7-26.2	6,902
Maldives	36.6 [10.3-59.9	41.3 [14.7-69.0]	23.3	20.7-25.9	26.4	24.8-28.1	5,587
Mali	17.9 [5.6-45.7]	23.8 [8.8-56.7]	21.8	20.7-22.9	23	22.2-24.0	601
Malta	70.7 [32.1-88.5]	73.1 [36.7-89.1]	27.7	26.0-29.4	27.1	24.6-29.5	19,727
Marshall Islands	43.5 [40.6-46.3]	55.7 [53.3-58.1]	29.4	28.2-30.6	31.4	29.8-33.0	2,838
Mauritania	40.0 [19.1-77.6]	47.6 [29.6-86.3]	22.6	21.2-24.0	26.3	25.0-27.5	896
Mauritius	38.2 [15.0-70.0]	39.1 [15.5-70.6]	25.1	23.8-26.4	26.1	24.4-27.6	6,951
Mexico	37.1 [14.8-70.0]	38.4 [15.5-71.7]	27.4	26.8-28.1	28.8	27.9-29.6	7,852
Micronesia	58.2 [55.2-61.1]	74.4 [70.2-78.6]	28.1	26.9-29.4	31.3	29.7-32.9	2,528
Moldova	25.4*	37.2*	24.3	21.7-26.	27.0	25.8-28.2	1,526
Monaco			.	.	.	.	172,676
Mongolia	9.3 [8.3-10.5]	9.5 [8.6-10.4]	24.9	24.4-25.4	25.7	25.1-26.4	1,690
Montenegro			26.8	24.4-29.1	25.7	22.6-28.7	6,569
Morocco	39.8*	48.7*	24.8	23.7-25.9	26.2	25.2-27.2	2,828
Mozambique	6.7 [5.4-8.2]	7.4 [6.3-8.7]	22	21.1-22.8	23.3	22.3-24.3	426
Myanmar	10.4 [9.3-11.6]	14.9 [13.9-15.9]	21.6	21.0-22.2	22.8	21.8-23.7	.
Namibia	51.9 [22.7-80.2]	65.1 [31.7-86.4]	22.7	21.8-23.8	25.2	24.3-26.0	4,096
Nepal	13.9 [4.5-38.3]	17.0 [5.9-44.1]	20.8	19.0-22.6	20.7	19.8-21.6	438
Netherlands	21.3 [8.2-52.5]	15.2 [5.2-43.6]	25.9	25.2-26.5	25.2	24.3-26.2	47,998
New Caledonia	52.8*	61.7*	.	.	.	.	36,311
New Zealand	45.0 [43.6-46.3]	50.4 [49.2-51.6]	27.8	27.1-28.4	27.4	26.5-28.3	29,352
Nicaragua	33.3*	43.3*	25.8	24.3-27.3	27.6	26.7-28.4	3,000
Niger	24.4 [22.1-26.8]	34.2 [31.6-36.9]	21.2	20.5-21.9	21.9	21.1-22.8	351
Nigeria	29.5*	36.5*	23	21.9-24.2	23.7	23.0-24.4	1,091
Norway	43.4 [18.7-75.5]	45.0 [18.9-76.6]	27	26.2-27.7	25.7	24.7-26.7	76,764
Oman			26.2	25.2-27.3	26.7	25.3-28.0	17,280
Pakistan	32.7 [12.7-65.7]	48.1 [20.1-76.6]	22.4	21.1-23.6	23.4	21.9-25.0	949
Palau			30.3	27.9-32.7	31.8	28.8-35.0	8,095
Panama	35.7*	44.6*	26.3	25.2-27.4	27.7	26.2-29.3	7,138
Papua New Guinea	17.2 [15.2-19.3]	21.5 [19.7-23.4]	25	24.3-25.8	25.8	24.6-27.0	1,181
Paraguay	40.7 [16.6-72.9]	42.0 [17.2-73.9]	25.6	23.0-28.2	25.9	22.5-29.4	2,245
Peru	16.8*	25.2*	24.8	24.2-25.4	26	25.4-26.7	4,412
Philippines	21.2 [7.6-53.6]	26.2 [9.8-60.4]	22.9	22.1-23.7	23.4	22.4-24.5	1,836
Poland	23.5 [8.7-56.8]	31.6 [12.3-66.1]	26.7	26.1-27.4	25.9	25.0-26.8	11,285
Portugal	47.5 [23.1-79.3]	54.4 [24.3-81.5]	26.7	25.8-27.6	26.2	25.1-27.4	22,027
Qatar			28.1	27.4-28.9	28.9	27.9-29.9	61,532
Romania	31.2 [11.9-65.5]	46.2 [18.5-76.4]	25.6	24.3-26.9	25.3	23.6-27.0	7,500
Russian Federation	22.7 [8.2-54.7]	18.8 [6.7-46.1]	26	25.5-26.6	27.2	26.5-27.9	8,615
Rwanda	2.7*	6.0*	22.7	20.2-25.2	22	21.0-23.0	510
Samoa	36.8 [34.3-39.3]	65.4 [62.7-68.1]	30.4	29.2-31.5	33.6	32.1-35.2	2,880
San Marino			.	.	.	.	60,895
Sao Tome and Principe	11.6 [9.3-14.1]	26.3 [24.5-28.4]	23.5	22.7-24.3	24.9	23.8-26.0	1,169
Saudi Arabia	61.5 [59.4-63.5]	76.2 [74.3-77.9]	27.9	27.2-28.6	29.6	28.7-30.4	13,901
Senegal	19.1 [6.1-48.0]	23.7 [8.0-55.0]	21.9	20.1-23.8	24.3	23.3-25.3	1,056
Serbia and Montenegro	63.2 [30.7-85.5]	73.3 [37.5-89.4]	27.2	26.5-27.9	25.4	24.4-26.3	5,484
Seychelles	22.4 [19.5-25.6]	22.4 [19.1-26.1]	25.5	24.6-26.5	27.9	26.7-29.1	9,028
Sierra Leone	16.2 [14.1-18.5]	23.6 [21.6-25.7]	22.5	21.9-23.2	23.9	23.2-24.7	323
Singapore			23.9	23.2-24.5	22.9	22.1-23.7	37,790
Slovakia	23.3 [8.2-55.6]	21.2 [7.5-53.1]	27.1	24.8-29.5	26.3	23.3-29.3	16,126
Slovenia	26.5 [9.6-59.9]	33.6 [13.2-68.0]	27.6	25.3-30.0	26.6	23.5-29.7	24,051
Solomon Islands	38.0 [35.3-40.8]	49.5 [47.0-52.0]	27.2	26.1-28.2	28.9	27.4-30.3	1,147
Somalia	22.6*	29.1*	22.1	19.8-24.4	22.6	19.7-25.6	333
South Africa	48.4 [46.0-50.9]	56.5 [54.4-58.5]	26.8	26.1-27.4	29.5	28.7-30.2	5,733
Spain	47.4 [20.1-78.1]	53.1 [23.6-81.0]	27.5	26.8-28.2	26.3	25.5-27.2	31,891
Sri Lanka	18.5 [17.5-19.5]	33.3 [32.2-34.5]	22	21.4-22.7	23.1	22.2-24.0	2,035
St. Kitts and Nevis	28.7 [24.8-32.8]	47.9 [44.5-51.2]	28.3	27.2-29.4	30.6	29.3-31.9	13,003
St. Lucia	42.5*	55.1*	24.9	23.0-26.7	27.7	25.3-30.0	6,423
St. Vincent and the Grenadines	44.4*	55.4*	25.7	23.4-27.9	27.9	24.8-30.9	6,398
Sudan	21.6*	28.8*	22.4	20.1-24.7	23.2	20.2-26.1	1,286
Suriname	37.1*	48.6*	25.7	23.3-28.0	28.1	25.2-31.1	6,254
Swaziland	65.9 [32.1-86.9]	72.1 [36.8-89.0]	23.2	20.8-25.6	28.5	27.4-29.6	2,513
Sweden	44.1 [18.9-75.9]	44.3 [19.3-75.8]	26.4	25.6-27.2	25.2	24.1-26.2	43,472
Switzerland	48.9*	48.5*	26.2	25.0-27.4	24.1	22.5-25.6	63,568
Syrian Arab Rep.	37.9*	48.1*	26.9	25.9-27.9	28.8	27.5-30.2	2,692
Tajikistan	38.0*	43.4*	23.8	21.4-26.1	23.9	22.0-25.8	734
Tanzania	26.3*	30.9*	22.4	21.0-23.8	22.2	21.0-23.4	503
Thailand	17.1 [16.3-17.8]	21.4 [20.6-22.1]	23	22.6-23.5	24.3	23.7-24.9	3,835
Timor-Leste	20.7*	27.4*	20.8	18.4-23.2	21.5	18.5-24.5	544
Togo	25.1*	32.6*	21.9	19.7-24.1	22.8	21.1-24.3	535
Tonga	31.8 [27.1-36.7]	51.9 [47.6-56.2]	31	29.9-32.0	34.2	32.8-35.7	3,011
Trinidad and Tobago	41.7*	52.4*	26.6	24.7-28.4	28.7	26.3-31.0	14,748
Tunisia	31.5 [12.4-63.8]	40.3 [17.0-72.1]	25.2	24.0-26.4	27.9	26.3-29.5	4,169
Turkey	49.5 [21.6-78.8]	62.5 [29.7-84.9]	26.7	26.3-27.1	28.3	27.6-28.9	8,554
Turkmenistan			25.2	22.9-27.6	24.7	23.0-26.4	3,745
Tuvalu							2,664
Uganda	24.1*	29.0*	22.5	20.0-25.0	22.4	21.5-23.4	488
Ukraine	20.4 [7.2-52.4]	16.3 [6.2-44.6]	25.5	23.0-27.9	26.3	24.5-28.1	2,545
United Arab Emirates	56.1 [25.8-82.9]	68.9 [34.6-88.3]	28	27.0-29.1	29.5	28.2-30.9	38,960
United Kingdom	58.0 [56.8-59.1]	68.6 [67.6-69.6]	27.4	26.9-27.9	27	26.3-27.6	35,163
USA	33.5 [31.9-35.2]	47.4 [45.7-49.1]	28.5	28.0-28.9	28.3	27.7-28.9	45,758
Uruguay	28.0 [24.5-31.6]	40.2 [37.5-42.9]	26.4	25.5-27.3	26.6	25.3-27.8	9,364
Uzbekistan	42.2*	47.9*	25.3	24.3-26.4	25.5	24.1-26.9	1,182
Vanuatu	38.4*	48.1*	26.8	25.7-27.9	28.5	27.2-29.8	2,526
Venezuela	48.6*	55.6*	27.5	26.2-28.7	28.2	26.6-29.8	11,490
Viet Nam	14.6 [4.5-39.5]	15.9 [5.2-41.8]	21	20.2-21.7	21.1	20.2-22.0	1,130
Yemen	48.6*	57.7*	24.5	22.2-26.7	26	23.0-29.2	1,130
Zambia	15.1 [4.8-39.5]	19.3 [6.6-48.2]	20.7	19.5-21.9	23.1	22.3-23.8	1,006
Zimbabwe	21.8 [7.9-51.8]	25.8 [9.5-58.9]	22.1	21.2-23.0	24.7	23.8-25.6	468

Table 3 presents the FAO/WHO univariate relationships between mean BMI and availability of 27 plant and animal food products in percent of total kcals of food available, percent “insufficient physical activity” and percapita GDP of the countries.

**Table 3:** Selected correlations of availability of foods and food and beverage groups to mean BMI from 167 Countries

**Food Groups and kcals**	**Mean % of kcals**	**SD**	**BMI 2008 r (*P*)**
Cereals	41.7	14.2	−0.46 (<0.0001)
Wheat	18.3	11.5	0.41 (<0.0001)
Rice	11.5	14.4	−0.41 (<0.0001)
Maize	7.56	10.7	−0.25 (<0.0001)
Vegetable oils	9.31	4.27	0.24 (<0.0001)
Soy	2.47	2.74	0.30 (<0.0001)
Rape and mustard seed	0.61	1.47	0.13 (0.0156)
Palm	1.57	2.34	−0.22 (<0.0001)
Sugar and sweets	9.90	4.70	0.60 (<0.0001)
Raw sugar equivalents	9.05	4.44	0.60 (<0.0001)
Meat and offals	7.46	4.48	0.54 (<0.0001)
Poultry	2.01	1.83	0.60 (<0.0001)
Cow	1.77	1.53	0.28 (<0.0001)
Pig	2.46	2.71	0.23 (<0.0001)
Sheep/Goat	0.64	1.14	0.20 (0.0002)
Offals	0.31	0.28	0.23 (<0.0001)
Roots and Tubers	7.37	9.24	−0.35 (<0.0001)
Potatoes	2.23	2.29	0.24 (<0.0001)
Cassava	2.85	6.78	−0.41 (<0.0001)
Milk, eggs, and Fish	7.58	4.59	0.44 (<0.0001)
Milk	3.99	3.35	0.26 (<0.0001)
Cheese	1.18	1.63	0.37 (<0.0001)
Eggs	0.76	0.61	0.41 (<0.0001)
Fruit	5.54	3.09	0.22 (<0.0001)
Animal fats	2.34	2.45	0.38 (<0.0001)
Pulses	2.30	2.40	−0.34 (<0.0001)
Other food groups	6.32	3.43	0.21 (0.0001)
Alcohol (g/d)	6.93	5.18	0.23 (<0.0001)
Kcals	2,612	717	0.28 (<0.0001)
% Insufficient physical activity excluding imputed estimates (n=224)	34.4	17.0	0.56 (<0.0001)
% Insufficient physical activity all (n=334)	35.7	15.6	0.59 (<0.0001)
Exercise (DCCT 1–4 scale)	1.97	0.48	−0.59 (<0.0001)
GDP 2009 (n=334)	11,261	16,558	0.35 (<0.0001)
Female gender	0.50	0.50	0.16 (0.0032)
BMI 2008	25.19	2.43	1.0

The FAO/WHO multiple regression derived formula relating mean BMI (dependent variable) to kcals, food group availability by percent of total kcals, exercise (DCCT 1–4 scale substituted for WHO “insufficient physical activity”), female gender, and percapita GDP of each country (independent variables) is below:

"Mean BMI (kg/m^2^) all (n=334) = 0.00131 kcals – 0.05980 cereals – 0.10346 all sugar and sweets + 0.04154 roots and tubers – 0.12157 cassava – 0.10796 + 0.02689 wheat + 0.03856 maize + 0.16379 sugar (raw) + 0.34879 poultry + 0.35408 sheep and goats – 1.21990 offals – 0.00002930 GDP percapita in 2009 + 1.89265 female gender – 1.14996 exercise (1–4 DCCT scale) + 24.598 (n=334 male and female cohorts, R^2^ = 0.74, P < 0.0001)."

In the univariate analysis, GDP percapita in 2009 is correlated with mean adult BMI (r=0.36, *P* < 0.0001). However, when GDP percapita in 2009 is included among the independent variables of the multiple regression, increasing GDP tends to decrease mean BMI. So GDP is not a factor in determining the level of adult BMI independent of macronutrient profile, exercise, and gender. Kcals percapita is not an important factor relating to mean adult BMI, accounting for only about 2% of the variance (R^2^ = 0.02 out of R^2^ = 0.74).

Breaking down the 334 cohorts to females, males, lower half of percapita GDP, and upper half of percapita GDP yields the following formulas:

"Mean BMI (kg/m^2^) females (n=167) = 0.00159 kcals + 0.10808 roots and tubers – 0.12987 pulses – 0.01983 rice + 0.02450 maize + 0.14742 sugar (raw) + 0.51772 poultry + 0.43560 sheep and goats + 1.41375 offals – 0.14879 cassava – 0.00003924 GDP percapita in 2009 – 1.30350 exercise + 24.8403; (R^2^ = 0.73, P < 0.0001);"

"Mean BMI (kg/m^2^) males (n=167) 0.00106 kcals – 0.07293 cereals – 0.08208 pulses + 0.02227 wheat + 0.02350 maize + 0.26662 poultry + 0.27629 sheep and goats – 1.17870 offals – 0.07866 cassava – 0.95467 exercise – 0.00001452 GDP percapita in 2009 + 25.94667; (R^2^ = 0.80, P < 0.0001)"

"Mean BMI (kg/m^2^) lower half countries in percapita GDP 2009 (n=166) = 0.00097484 kcals – 0.08496 cereals – 0.06629 vegetable oil + 0.34149 animal fat – 0.16668 pulses + 0.02239 wheat + 0.02516 maize + 0.17003 soy oil – 1.08248 rape oil + 0.54829 sheep and goats – 3.84885 offals – 0.09109 cassava – 0.11750 milk + 1.10932 cheese + 1.80429 female – 1.27746 exercise + 28.7249; (R^2^ = 0.81, P < 0.0001)"

"Mean BMI (kg/m^2^) upper GDP 2009 half countries in percapita GDP (n=166) = 0.03909 cereals – 0.08294 roots and tubers – 0.06886 rice + 0.07593 sugar (raw) + 0.32613 poultry + 0.18367 potatoes + 0.24919 sheep and goats – 0.89450 offals 0.67179 eggs – 0.71995 exercise + 25.78858; (R^2^ = 0.50, P < 0.0001);"

Table 4 shows the fairly strong correlations of the multiple regression derived formula predicting mean BMI in 2008 (n=334 female and male cohorts) with similarly derived formulas relating to single sex cohorts (female and male) and percapita 2009 GDP (below and above the median). These strong correlations suggest that the food group profiles and exercise levels determine the mean adult BMI virtually independently from gender or country per capita GDP.

**Table 4:** Correlations of FAO/WHO mean female and male cohort BMIs in 2008 with formulas predicting mean BMI

	**BMI 2008 r (*P*)**	**BMI 2008 formula all cohorts r (*P*)**	**BMI 2008 formula females r (*P*)**	**BMI 2008 formula males r (*P*)**	**BMI 2008 formula lower half GDP 2009 r (*P*)**	**BMI 2008 formula upper half GDP 2009 r (*P*)**
BMI 2008 formula all cohorts	0.86 (< 0.0001)	1.0	0.854 (< 0.0001)	0.88 (< 0.0001)	0.75 (< 0.0001)	0.73 (< 0.0001)
BMI 2008 formula females	0.74 (< 0.0001)	0.85 (< 0.0001)	1.0	0.90 (< 0.0001)	0.56 (< 0.0001)	0.72 (< 0.0001)
BMI 2008 formula males	0.76 (< 0.0001)	0.88 (< 0.0001)	0.90 (< 0.0001)	1.0	0.74 (< 0.0001)	0.66 (< 0.0001)
BMI 2008 formula lower half GDP 2009	0.65 (< 0.0001)	075 (< 0.0001)	0.56 (< 0.0001)	0.74 (< 0.0001)	1.0	0.50 (< 0.0001)
BMI 2008 formula upper half GDP 2009	0.63 (< 0.0001)	0.73 (< 0.0001)	0.72 (< 0.0001)	0.66 (< 0.0001)	0.50 (< 0.0001)	1.0

Table 5 shows the template for using the USDA Nutrient Database for Standard Reference, Release 24 [19] to convert FAO food group data to macronutrient profiles for each country. As an example of using this template, Table 6 demonstrates the breakdown of food availability by food group in the USA transformed into the macronutrient profile. Table 7 shows the resulting template derived macronutrient availability data in percent of kcals (g per 1,000 kcals for dietary fiber) for male and female cohorts from the 167 countries.

**Table 5:** Template used for converting FAO food group availability data in percent of total kcals to macronutrients (g)

**FAO Food groups**	**Kcals**	**Protein**	**Carbs**	**Dietary fiber**	**PUFA**	**MUFA**	**SFA**	**Total fat**	**Alcohol**
Cereals	100	2.58	19.2	1.61	0.64	0.56	0.29	1.42	0.00
Vegetable oil	100	0.00	0.00	0.00	3.09	4.27	3.24	11.1	0.00
Sugar and sweeteners	100	0.00	25.0	0.00	0.00	0.00	0.00	0.00	0.00
Meats	100	6.40	0.33	0.00	0.70	4.14	2.71	8.12	0.00
Roots and tubers	100	1.53	23.2	2.87	0.07	0.02	0.04	0.13	0.00
Milk, eggs, fish	100	9.74	4.00	0.00	0.88	2.05	1.69	5.00	0.00
Fruits	100	1.11	21.3	2.63	0.21	0.89	0.18	1.15	0.00
Animal fat	100	0.00	0.00	0.00	1.20	5.46	3.74	11.1	0.00
Pulses	100	5.64	12.9	4.23	1.08	1.49	0.42	2.87	0.00
Alcohol with the associated carbohydrates from wine (fruit) and beer (cereals)	100	0.00	12.1	0.00	0.00	0.00	0.00	0.00	14.30

**Table 6:** Converting FAO food group availability data in kcals to macronutrients (g) for the USA

**FAO Food groups**	**Kcals**	**Protein**	**Carbs**	**Dietary fiber**	**PUFA**	**MUFA**	**SFA**	**Total fat**	**Alcohol**
Cereals	821	22.0	164	13.2	5.50	4.60	2.38	12.2	0.00
Vegetable oil	667	0.00	0.00	0.00	19.5	25.9	20.0	74.3	0.00
Sugar and sweeteners	638	0.00	161	0.00	0.00	0.00	0.00	0.00	0.00
Meats	455	28.5	1.50	0.00	3.46	19.8	13.0	36.2	0.00
Roots and tubers	96	1.51	22.8	2.76	0.06	0.01	0.03	0.12	0.00
Milk, eggs, fish	466	45.4	15.0	0.00	4.61	10.4	8.67	23.6	0.00
Fruits	193	2.40	46.2	5.09	0.44	1.82	0.37	2.47	0.00
Animal fat	109	0.00	0.00	0.00	1.50	6.68	4.59	11.9	0.00
Pulses	41	2.43	5.55	1.75	0.51	0.68	0.19	1.24	0.00
Alcohol	124	0.00	5.56	0.00	0.00	0.00	0.00	0.00	14.2
Totals	3,659	102	422	22.8	35.6	69.9	49.3	162	14.2

**Table 7:** **Macronutrient availability as percent of total available kcals from 167 countries**[14]**(Fiber is in g/1,000 kcals/d)**

**Country**	**Kcals**	**Protein**	**Carbs**	**Fiber**	**PUFA**	**MUFA**	**SFA**	**Total fat**
Afghanistan			.	.	.	.	.	.
Albania	2,832	11.2	61.1	12.9	6.0	11.8	7.3	25.8
Algeria	3,075	10.3	64.2	12.8	7.4	10.5	6.8	25.2
Andorra	.	.	.	.	.	.	.	.
Angola	1,918	8.7	66.2	15.6	6.3	9.5	6.3	22.8
Antigua and Barbuda	2,270	11.4	52.2	9.5	6.9	15.4	10.0	33.7
Argentina	2,967	10.5	54.5	9.0	6.6	14.7	9.6	32.2
Armenia	2,266	11.2	62.3	12.3	5.7	10.3	6.6	23.2
Australia	3,030	10.2	46.9	7.9	8.1	18.3	12.3	40.6
Austria	3,494	9.8	46.8	7.9	7.8	18.2	12.3	40.2
Azerbaijan	2,937	10.5	65.7	13.2	5.8	9.3	5.8	21.3
Bahamas	2,569	12.2	49.6	7.7	6.2	16.3	10.8	35.0
Bahrain	.	.	.	.	.	.	.	.
Bangladesh	2,220	9.7	71.3	15.1	6.8	7.6	4.5	18.9
Barbados	2,824	10.3	59.0	10.1	6.4	13.1	8.4	28.9
Belarus	3,038	9.4	58.1	11.8	6.1	13.3	8.6	29.1
Belgium	3,471	7.9	49.2	8.6	8.3	18.3	12.3	40.8
Belize	2,562	12.9	58.3	10.2	5.8	12.4	8.0	27.2
Benin	2,332	9.2	70.7	18.9	5.9	8.0	5.2	19.5
Bermuda	.	.	.	.	.	.	.	.
Bhutan	.	.	.	.	.	.	.	.
Bolivia	2,010	11.6	61.8	11.1	5.5	11.2	7.2	24.8
Bosnia and Herzegovina	2,806	11.2	62.9	13.9	6.4	10.2	6.4	23.5
Botswana	2,172	8.9	65.3	14.1	6.6	10.1	6.2	23.3
Brazil	2,991	10.4	54.1	10.9	7.7	14.7	9.6	33.3
Brunei Darussalam	2,787	10.2	62.0	11.5	6.8	12.0	7.7	27.2
Bulgaria	2,647	8.6	54.7	10.4	8.3	14.4	9.5	33.4
Burkina Faso	2,446	10.9	66.7	15.1	6.8	8.6	5.0	20.4
Burundi	1,625	15.2	63.3	22.0	4.8	7.6	4.5	17.3
Cambodia	2,178	10.8	68.8	14.4	5.8	8.2	4.8	18.9
Cameroon	2,124	11.8	61.2	15.4	7.0	10.2	6.7	24.6
Canada	3,337	9.0	48.8	8.5	8.5	17.8	12.0	40.1
Cape Verde	2,463	10.0	61.4	12.6	6.8	12.0	7.6	27.2
Central African Republic	1,818	9.6	59.5	15.7	7.3	12.6	8.6	29.7
Chad	1,725	10.0	68.1	16.1	6.4	8.5	5.1	20.2
Chile	2,925	10.2	56.4	9.9	7.1	13.9	9.1	31.4
China	2,851	13.3	55.2	11.9	6.7	13.5	8.8	30.1
Colombia	2,622	10.1	61.4	10.6	6.6	11.6	7.7	26.8
Comoros	1,727	11.5	62.3	16.2	7.6	10.9	7.0	26.1
Congo	2,421	8.9	64.5	15.9	6.9	10.3	7.2	25.4
Congo, Dem Republic	1,511	8.2	75.5	21.5	4.4	5.8	4.1	14.7
Costa Rica	2,718	8.6	62.2	10.9	7.0	12.1	7.8	27.7
Cote d'Ivoire	2,399	9.7	64.7	15.8	6.7	9.5	6.8	23.8
Croatia	2,802	8.4	58.4	10.3	7.1	12.9	8.5	29.5
Cuba	3,213	11.4	65.8	13.2	5.8	9.4	5.9	21.6
Cyprus	2,920	10.7	52.7	9.4	7.3	15.5	10.1	34.3
Czech Republic	3,053	8.8	52.8	9.4	7.5	15.4	10.2	34.6
Denmark	3,166	9.7	51.2	8.8	5.9	17.3	11.2	36.2
Djibouti	.	.	.	.	.	.	.	.
Dominica	2,836	12.4	58.4	11.1	5.6	12.4	8.1	27.2
Dominican Republic	2,157	10.5	52.7	9.2	8.6	14.8	10.0	34.7
Ecuador	2,243	12.7	48.3	9.5	8.9	16.9	11.6	39.0
Egypt	3,063	11.8	66.6	13.2	6.3	9.1	5.7	21.5
El Salvador	2,497	10.0	67.4	13.1	6.0	9.5	5.7	21.6
Equatorial guinea	.	.	.	.	.	.	.	.
Eritrea	1,566	10.1	65.0	15.8	7.9	10.1	6.1	24.2
Estonia	2,870	8.7	61.2	11.0	5.5	12.2	7.7	26.2
Ethiopia	1,935	10.6	72.0	18.1	5.5	6.9	3.8	16.0
Faeroe Islands	.	.	.	.	.	.	.	.
Fiji	2,789	9.3	61.3	12.4	6.9	12.8	8.2	28.8
Finland	3,039	10.3	53.7	10.7	6.4	15.5	9.9	33.1
France	3,401	9.7	48.2	9.3	7.7	18.0	11.8	39.2
French Polynesia	2,628	13.3	43.9	8.2	8.1	18.0	12.3	40.3
Gabon	2,523	13.2	58.6	12.8	5.9	11.1	7.6	25.5
Gambia	2,222	7.2	60.4	11.1	9.6	12.5	8.5	31.4
Georgia	2,753	10.3	65.6	13.5	5.8	10.1	6.1	22.4
Germany	3,277	8.6	51.3	8.9	7.4	17.0	11.3	37.4
Ghana	2,683	10.9	71.1	18.0	5.0	7.3	5.2	18.1
Greece	3,478	10.8	50.9	10.1	8.7	15.6	10.5	36.1
Greenland	.	.	.	.	.	.	.	.
Grenada	.	.	.	.	.	.	.	.
Guatemala	2,121	9.6	66.3	11.7	6.6	9.7	6.1	22.8
Guinea	2,459	11.2	60.5	13.5	8.2	11.3	7.8	28.1
Guinea-Bissau	2,242	9.9	62.2	13.2	7.9	10.9	7.3	26.8
Guyana	2,579	10.1	65.9	12.8	5.8	9.4	5.8	21.4
Haiti	1,777	11.0	64.9	13.4	6.1	9.2	5.8	21.5
Honduras	2,572	10.1	62.3	11.2	6.9	11.4	7.3	26.4
Hong Kong	.	.	.	.	.	.	.	.
Hungary	3,276	8.8	47.2	8.3	7.9	18.3	12.3	40.5
Iceland	3,093	10.0	56.1	10.8	5.5	15.8	9.8	32.5
India	2,238	9.6	64.2	13.1	7.6	10.5	6.7	25.3
Indonesia	2,376	9.6	69.9	14.3	6.5	8.4	5.2	20.3
Iran	2,998	10.1	66.9	14.0	6.3	10.0	6.1	22.7
Iraq	.	.	.	.	.	.	.	.
Ireland	3,285	13.0	47.0	7.7	7.4	16.4	11.3	37.0
Isle of Man	.	.	.	.	.	.	.	.
Israel	3,353	11.0	48.4	9.7	9.5	17.3	11.7	40.1
Italy	3,631	11.6	45.1	9.6	9.4	18.0	12.1	41.2
Jamaica	2,720	10.3	58.6	9.6	6.9	13.1	8.7	29.8
Japan	2,553	12.1	53.3	9.2	8.0	13.7	9.4	32.4
Jordan	2,898	9.6	59.5	10.5	8.5	12.8	8.6	30.8
Kazakhstan	3,327	13.0	55.3	9.8	6.9	12.7	8.7	29.4
Kenya	2,040	11.4	63.7	14.4	6.8	10.1	6.2	23.5
Kiribati	.	.	.	.	.	.	.	.
Korea, Dem. Republic	2,073	10.6	67.1	16.6	6.5	9.2	5.3	21.2
Korea, Republic	2,832	9.9	58.9	10.6	7.7	13.1	8.6	30.3
Kosovo	.	.	.	.	.	.	.	.
Kuwait	2,926	10.6	52.7	9.4	8.9	15.7	10.6	36.6
Kyrgyz Republic	2,651	13.1	63.8	13.3	5.8	9.4	6.0	21.7
Laos	2,131	10.8	69.2	15.1	5.6	7.7	4.4	17.7
Latvia	2,875	12.0	48.2	8.3	7.7	16.1	11.2	36.8
Lebanon	2,880	10.2	56.4	11.5	8.0	14.2	9.4	32.8
Lesotho	2,477	10.2	72.2	14.7	5.6	6.7	3.8	16.0
Liberia	2,110	7.2	62.2	15.6	8.6	11.6	8.0	29.0
Libya	3,001	9.7	58.3	10.6	8.8	13.2	9.0	32.0
Liechtenstein	.	.	.	.	.	.	.	.
Lithuania	3,264	12.5	51.8	9.5	7.0	14.3	9.7	32.4
Luxembourg	3,198	16.3	39.2	6.7	7.6	18.9	12.9	41.6
Macao SAR, China	.	.	.	.	.	.	.	.
Macedonia, FYR	2,781	9.5	55.3	10.3	8.2	15.4	10.3	35.2
Madagascar	2,108	10.1	72.2	17.2	5.4	7.3	4.4	17.2
Malawi	2,069	9.5	75.6	18.8	5.1	6.1	3.3	14.3
Malaysia	2,789	11.3	56.4	9.7	8.1	13.6	9.2	32.1
Maldives	.	.	.	.	.	.	.	.
Mali	2,541	11.5	64.3	14.0	7.3	10.0	6.2	23.9
Malta	3,345	12.2	56.9	9.5	6.1	13.6	9.0	30.0
Marshall Islands	.	.	.	.	.	.	.	.
Mauritania	2,802	12.1	58.1	10.1	7.9	12.5	8.4	29.8
Mauritius	2,828	10.8	57.5	10.7	8.3	12.9	8.6	30.8
Mexico	3,183	11.3	58.8	10.5	6.7	12.4	8.0	28.0
Micronesia	.	.	.	.	.	.	.	.
Moldova	2,770	11.7	60.0	11.1	6.0	10.2	6.7	23.6
Monaco	.	.	.	.	.	.	.	.
Mongolia	2,198	14.3	49.8	9.0	7.0	15.8	10.6	34.9
Montenegro	.	.	.	.	.	.	.	.
Morocco	3,181	9.2	68.4	13.2	6.7	9.3	5.8	22.1
Mozambique	2,046	8.4	73.3	19.2	5.8	7.1	4.5	17.6
Myanmar	2,331	10.9	63.3	14.2	7.6	10.9	6.7	25.7
Namibia	2,287	10.6	63.4	13.2	5.9	10.0	6.5	23.1
Nepal	2,266	10.4	65.6	15.0	7.4	9.6	6.0	23.2
Netherlands	3,060	12.9	45.6	7.1	8.0	17.2	12.0	39.2
New Caledonia	2,515	12.3	47.9	8.8	8.4	16.6	11.4	38.0
New Zealand	3,024	11.2	50.9	7.7	6.3	16.5	11.0	35.6
Nicaragua	2,377	10.6	64.1	12.2	7.0	10.0	6.3	23.7
Niger	2,160	11.2	67.0	16.6	7.1	9.2	5.3	21.6
Nigeria	2,571	8.0	65.1	16.1	7.3	9.5	6.2	23.6
Norway	3,273	12.4	47.1	7.6	7.9	17.2	11.9	38.9
Oman	.	.	.	.	.	.	.	.
Pakistan	2,210	11.4	57.2	10.3	8.0	13.3	9.0	31.4
Palau	.	.	.	.	.	.	.	.
Panama	2,368	11.3	57.2	10.3	7.0	12.9	8.5	29.5
Papua New Guinea	.	.	.	.	.	.	.	.
Paraguay	2,429	10.8	54.9	12.3	8.1	14.8	10.0	34.4
Peru	2,291	10.5	68.7	15.2	5.7	9.1	5.6	24.3
Philippines	2,467	10.6	64.7	12.2	5.7	10.3	6.4	23.0
Poland	3,307	11.0	53.0	9.2	6.8	14.8	10.0	33.2
Portugal	3,329	11.9	46.1	8.7	8.0	17.3	11.8	38.9
Qatar	.	.	.	.	.	.	.	.
Romania	3,416	12.8	53.1	10.2	7.3	13.4	9.1	31.0
Russian Federation	3,154	11.5	55.8	9.8	6.7	12.7	8.6	29.2
Rwanda	1,990	8.8	74.3	24.5	4.0	6.5	3.1	13.5
Samoa	2,333	11.9	51.2	9.9	6.1	16.7	11.0	35.5
San Marino	.	.	.	.	.	.	.	.
Sao Tome and Principe	2,317	8.8	68.0	15.9	5.9	8.8	5.4	20.5
Saudi Arabia	3,056	10.3	60.1	11.5	7.7	12.8	8.3	29.6
Senegal	2,225	9.9	61.1	12.3	8.7	11.7	7.8	28.9
Serbia and Montenegro	2,576	10.4	36.1	6.9	6.6	16.0	10.7	34.9
Seychelles	2,248	11.9	56.2	10.5	7.0	12.4	8.2	28.7
Sierra Leone	1,987	9.2	60.3	15.0	8.5	11.0	7.1	27.1
Singapore	.	.	.	.	.	.	.	.
Slovakia	2,732	9.5	49.6	8.3	7.9	16.6	11.3	37.5
Slovenia	3,072	12.0	45.3	9.0	8.1	17.5	11.8	39.2
Solomon Islands	2,196	9.7	75.2	20.0	4.4	6.2	3.8	15.7
Somalia	1,728	15.9	54.8	8.3	6.3	12.7	8.8	29.1
South Africa	2,857	10.1	59.3	10.6	7.5	12.0	7.9	28.2
Spain	3,067	11.9	41.0	7.4	9.8	19.1	13.3	44.4
Sri Lanka	2,050	10.2	73.4	13.8	5.4	6.8	3.9	16.1
St. Kitts and Nevis	2,267	12.9	50.0	7.6	6.9	15.3	10.3	34.2
St. Lucia	2,553	13.5	51.6	9.2	5.9	14.7	9.6	31.6
St. Vincent and the Grenadines	2,630	10.8	58.9	10.2	6.6	12.8	8.4	28.9
Sudan	2,198	15.0	57.6	10.8	6.7	11.4	7.6	26.6
Suriname	2,352	8.7	60.5	9.5	7.6	12.3	8.2	29.0
Swaziland	2,157	11.4	63.8	11.9	5.8	10.1	6.4	23.0
Sweden	2,987	13.3	45.4	6.9	7.8	17.2	12.0	38.9
Switzerland	3,213	12.1	45.1	6.0	7.9	18.0	12.5	40.4
Syrian Arab Republic	2,904	10.3	58.4	10.9	8.2	13.1	8.7	31.0
Tajikistan	2,121	10.0	63.6	12.4	7.6	10.4	6.8	25.3
Tanzania	1,982	9.8	68.5	16.7	6.1	8.1	5.0	19.3
Thailand	2,293	10.3	64.2	11.3	6.2	10.1	6.4	23.3
Timor-Leste	1,963	11.7	68.6	15.7	5.4	8.5	5.1	19.4
Togo	2,036	8.8	70.4	17.9	6.5	8.1	5.2	20.1
Tonga	.	.	.	.	.	.	.	.
Trinidad and Tobago	2,603	9.9	59.5	9.3	7.0	12.5	8.3	28.9
Tunisia	3,214	9.7	60.0	11.8	8.5	12.4	8.2	30.0
Turkey	3,368	10.2	59.9	12.7	8.3	12.2	8.0	29.3
Turkmenistan	.	.	.	.	.	.	.	.
Tuvalu	.	.	.	.	.	.	.	.
Uganda	1,998	9.0	67.8	20.2	5.2	8.8	4.9	19.1
Ukraine	3,175	10.7	57.7	10.2	6.7	12.1	8.2	28.1
United Arab Emirates	2,986	12.4	59.0	11.4	6.7	12.7	8.1	28.4
United Kingdom	3,246	12.5	46.5	8.2	7.7	16.9	11.6	38.1
USA	3,565	11.4	45.8	6.4	9.0	17.6	12.4	40.9
Uruguay	2,741	12.5	55.0	9.6	6.6	13.6	9.0	30.4
Uzbekistan	2,504	12.6	56.2	11.6	8.1	12.7	8.5	30.3
Vanuatu	2,217	10.4	65.9	15.0	5.4	10.5	6.8	23.4
Venezuela	2,496	9.6	55.5	9.3	8.2	13.9	9.4	32.6
Viet Nam	2,678	11.5	65.0	13.4	5.9	10.1	6.1	22.5
Yemen	1,999	9.5	65.5	11.9	7.4	10.4	6.7	25.0
Zambia	1,855	9.4	70.4	15.2	6.0	7.7	4.8	18.7
Zimbabwe	2,070	8.6	64.0	11.6	7.5	10.7	6.9	25.7

Table 8 presents the univariate relationships between mean BMI and macronutrients available as g and percentages of total kcals of food available in the 167 countries.

**Table 8:** Correlations of macronutrient availability with mean BMI 2008 for female and male cohorts from 167 countries

**Macronutrients and kcals available**	**Mean intake (90% CI)**	**BMI 2008 r (*P*)**
Kcals	2,613 (1,855-3,353)	0.28 (<0.0001)
Protein g	70.2 (38.5-111)	0.30 (<0.0001)
Protein % of kcals	10.7 (8.60-13.3)	0.13 (0.0184)
Carbohydrate g	382 (248–536)	0.04 (0.44)
Carbohydrate % of kcals	59.3 (45.8-72.0)	−0.52 (<0.0001)
Dietary fiber g	30.4 (18.2-46.2)	−0.27 (<0.0001)
Dietary fiber g per 1,000 kcals	12.0 (7.60-18.1)	−0.60 (<0.0001)
PUFA g	20.4 (10.5-33.9)	0.31 (<0.0001)
PUFA % of kcals	6.97 (5.39-8.84)	0.20 (<0.0001)
MUFA g	36.8 (14.7-72.9)	0.46 (<0.0001)
MUFA % of kcals	12.3 (7.28-18.0)	0.55 (<0.0001)
SFA g	24.2 (8.62-49.4)	0.47 (<0.0001)
SFA % of kcals	8.06 (4.42-12.3)	0.54 (<0.0001)
Total fat g	84.5 (34.0-164)	0.45 (<0.0001)
Total fat % of kcals	28.3 (17.3-40.4)	0.52 (<0.0001)
Total fat g – PUFA g	64.0 (44.5-130)	0.47 (<0.0001)
Total fat % of kcals – PUFA % of kcals	21.3 (10.3-31.6)	0.55 (<0.0001)
Alcohol g	6.92 (0.27-16.7)	0.23 (<0.0001)

The multiple regression derived FAO/WHO formula relating mean adult BMI to macronutrient availability and exercise (DCCT 1–4 scale) is below:

"Mean BMI (kg/m^2^) for female and male cohorts (n=334) = 0.07710 alcohol (g) + 0.11947 carbohydrates (% of kcals) – 0.30486 dietary fiber (g/1000 kcals) + 0.19733 total fat (% of kcals) – 0.68567 PUFA (% of kcals) – 2.14356 exercise (DCCT 1–4 scale) + 24.64; R^2^=0.55"

In the initial FAO/WHO multiple regression analysis, protein, kcals and MUFA appeared in the formula, and carbohydrates, total fat, PUFA, SFA and alcohol did not appear. To increase the spectrum of the macronutrient profile covered by the formula, protein (about 10% of kcals), MUFA, SFA, and kcals were omitted, resulting in carbohydrates (about 60% of kcals), total fat, PUFA, alcohol (mean=6.66 g/day consumed) appearing. The b-weight of the alcohol (g) variable was doubled because alcohol did not appear in the DCCT/EDIC macronutrient and exercise formula. The multiple correlations coefficient was almost identical whether protein, MUFA, SFA, and kcals were or were not included as independent variables (R^2^=0.56 versus R^2^=0.54).

Formulas of sub cohorts broken down by gender and percapita GDP are below:

"Mean BMI (kg/m^2^) female cohorts only (n=167) = 0.00147 kcals + 0.06522 carbohydrates (% of kcals) – 0.37905 dietary fiber (g/1,000 kcals) – 0.46653 PUFA −2.25919 exercise + 30.63; R^2^=0.53"

"Mean BMI (kg/m^2^) male cohorts only (n=167) = 0.00142 kcals + 0.08247 carbohydrates (% of kcals) + 0.30007 total fat – 0.85814 PUFA % of kcals – 1.44131 exercise (DCCT 1–4 scale) + 15.95; R^2^ =0.71"

"Mean BMI (kg/m^2^) percapita GDP below the median (n=166) = 0.05741 alcohol (g/day) + 0.4099 carbohydrates – 0.26010 dietary fiber (g/1,000 kcals) + 0.67133 total fat – 1.50540 PUFA – 2.18068 exercise (DCCT 1–4 scale) – 0.06; R^2^=0.57"

"Mean BMI (kg/m^2^) percapita GDP above the median (n=166) = 0.08058 carbohydrates (% of kcals) – 0.36953 dietary fiber (g/1,000 kcals) – 0.98044 exercise (DCCT 1–4 scale) + 27.59; R^2^=0.23"

Table 9 shows the strong correlations of the BMI in 2008 with the various BMI prediction formulas and the correlations of the formulas with each other.

**Table 9:** Correlations of BMI 2008 with macronutrient profiles and exercise (DCCT 1–4 scale) N=334

	**BMI 2008 r (*P*)**	**BMI 2008 formula all cohorts r (*P*)**	**BMI 2008 formula females r (*P*)**	**BMI 2008 formula males r (*P*)**	**BMI 2008 formula lower half GDP 2009 r (*P*)**	**BMI 2008 formula upper half GDP 2009 r (*P*)**
BMI 2008 formula all cohorts	0.74 (< 0.0001)	1.0	0.85 (< 0.0001)	0.83 (< 0.0001)	0.93 (< 0.0001)	0.89 (< 0.0001)
BMI 2008 formula females	0.65 (< 0.0001)	0.85 (< 0.0001)	1.0	0.87 (< 0.0001)	0.84 (< 0.0001)	0.88 (< 0.0001)
BMI 2008 formula males	0.63 (< 0.0001)	0.83 (< 0.0001)	0.87 (< 0.0001)	1.0	0.75 (< 0.0001)	0.68 (< 0.0001)
BMI 2008 formula lower half GDP 2009	0.69 (< 0.0001)	0.95 (< 0.0001)	0.84 (< 0.0001)	0.75 (< 0.0001)	1.0	0.79 (< 0.0001)
BMI 2008 formula upper half GDP 2009	0.66 (< 0.0001)	0.89 (< 0.0001)	0.88 (< 0.0001)	0.87 (< 0.0001)	0.89 (< 0.0001)	1.0

### DCCT/EDIC analysis

Table 10 shows the baseline and on study age, sex, BMI, HbA1c, and exercise levels of the 1,055 DCCT/EDIC participants with average HbA1c levels < 9.5. Table 11 compares the Pearson correlations of macronutrients with BMI change/year.

**Table 10:** Baseline characteristics and lifestyle factor data of 1,055 DCCT participants with HbA1c ≤ 9.5

**Variable**	**Baseline mean [90% CI]**	**On trial results mean [90% CI]**	**Correlation with BMI change/ year r (*P*)**
Age (years)	27.4 [15.0 to 38.0]	42.8 [31.2 to 53.2] (closeout)	−0.09 (0.0025)
BMI	23.4 [19.4 to 27.9]	27.6 [21.6 to 35.7] (closeout)	−0.02 (0.52) (re baseline BMI)
BMI change/year	-	0.27 [−0.06 to 0.70]	1.0
Female %	46.9 [−]		−0.04 (0.24)
Exercise: 1–4 scale	-	1.83 [1.00 to 2.67]	−0.10 (0.0012)
HbA1c - weighted mean of DCCT and EDIC	-	7.85 [6.43 to 9.23]	0.02 (0.42)

**Table 11:** Nutrient intake related to BMI change/year for DCCT/EDIC mean follow up of 16.4 years

**Variable**	**Mean intake in g or % kcals [90% CI]**	**BMI change/year vs. % kcals r**	***P***
Food energy (kcals)	2,297	0.04	0.22
Protein g	102 [62.1-160]	0.03	0.34
Protein % of kcals	17.9 [14.5-21.7 ]	−0.02	0.47
Carbohydrates g	264 [159–405]	0.02	0.46
Carbohydrates % of kcals	46.5 [37.3-55.9]	−0.03	0.40
Dietary fiber g	24.3 [13.6-39.7]	−0.07	0.0263
Dietary fiber g /1000 kcals	10.9 [6.79-16.2]	−0.12	0.0002
PUFA g	19.1 [9.05-33.2]	−0.00	0.92
PUFA % of kcals	7.45 [4.97-10.3]	−0.06	0.06
MUFA g	35.5 [16.5-62.9]	0.06	0.0416
MUFA % of kcals	13.7 [9.50-17.5]	0.07	0.0318
SFA g	32.3 [15.0-58.7]	0.06	0.07
SFA % of kcals	12.4 [8.46-16.4]	0.06	0.0469
Trans fat g (Total fat – SFA–MUFA–PUFA g)	6.81 [3.84-11.0]	0.04	0.25
Trans fat % kcals (Total fat – SFA–MUFA–-PUFA % kcals)	2.66 [2.21-3.12]	0.00	0.88
Total Fat g	93.7 [45.9-165]	0.05	0.11
Total Fat % of kcals	36.2 [26.6-45.1]	0.04	0.22
Total fat – PUFA g	74.6 [36.1-133]	0.06	0.06
Total fat – PUFA % of kcals	28.8 [20.8-36.2]	0.07	0.0331
Alcohol g	3.35 [0.00-15.9]	−0.04	0.15
Alcohol % of kcals	0.96 [0.00-4.16]	−0.06	0.0549

The DCCT/EDIC participant BMI change/year correlated inversely with baseline age (r = −0.10, *P* = 0.0012), however, age and caloric intake were also negatively correlated (r = −0.15, *P* < 0.0001). While BMI change/year did not correlate significantly with sex (r = 0.04, *P* = 0.24), the caloric intake of males exceeded that of females by 51% (mean [90% CI] = 2,732 [1,869 to 3,827] kcals versus 1,804 [1,231 to 2,527] kcals). As with the FAO/WHO analysis, to compensate for the marked influence of age and sex on macronutrient intake while there was an inverse correlation of age and no influence of sex on BMI change/year, the percentages of the kcals contributed by each macronutrient were evaluated in addition to grams of each macronutrient (e.g., (protein (g) × 4 kcals/g/ kcals) × 100 = percentage of kcals as protein). Dietary fiber was expressed as g/1,000 kcals. Since the sum of SFA % kcals, MUFA % kcals, and trans fats % kcals (i.e., total fat – PUFA) directly correlated with BMI change/year in the univariate analysis (r = 0.07, *P* = 0.0331, from Table 11), these three macronutrients were combined as a single variable. This facilitated the generation of the following formula including about 97% of the macronutrient profile:

"BMI change/year DCCT/EDIC formula= (0.00898 protein (% of kcals) + 0.01063 carbohydrates (% of kcals) – 0.01319 dietary fiber (g/1,000 kcal) + 0.00973 (total fats – PUFA) (% of kcals) – 0.04468 exercise (1–4 scale)) * 0.545 – 0.119; R^2^ = 0.03, P < 0.0001)."

The initial formula was multiplied by 0.545 to adjust the SD of the formula from 0.416 to the SD of the DCCT/EDIC subjects (0.227). A constant adjusted the mean output to the mean BMI change/year of the DCCT/EDIC subjects (0.268).

### Comparisons of FAO/WHO and DCCT/EDIC formulas

To facilitate the comparison of predictions of future BMIs from the FAO/WHO and DCCT/EDIC formulas, both formulas were adjusted, as described in the methods, to convert macronutrient variables from percent of total kcals to grams of macronutrients, as would enable the use of the interactive web-based future BMI prediction tool. The adjustment also involved changing the output of the FAO/WHO formula from “mean BMI” to “BMI change/year.” This conversion was made by designating the mean BMI of 20 year old people in the USA (mean BMI=22.4, according to the Center for Disease Control [28]) as the baseline adult BMI. US Census Bureau data were used to estimate the median age of adults over 25 years old in the USA (median age of adults > 25 years old = 48.9 years old [29,30]). For adults ≥ 25 years old, median BMI in the USA in 2008 was 28.4 (averaging females and males, Table 2). Using these figures, the average USA person had a BMI change/year over baseline adult BMI = 0.123 BMI change/year (28.4 – 22.4 = 6.0 BMI units above baseline; 6.0/48.9 (48.9 = median age of adults > 25 years old) = 0.123 BMI change/year). To derive the SD (σ) of the BMI change/year above the baseline adult BMI, the BMI change/year for obese people was calculated (BMI ≥ 30 – 22.4 ≥ 7.6 BMI units above baseline; 7.6/48.9 = 0.155 BMI change/year) and the USA incidence of adult obesity (33%) ascertained from the literature [31]. These values were plugged into the formula for a normal distribution [30]: $p\left(x\right)=\frac{1}{\sqrt[{\sigma }]{2\pi }}\exp\left(-\frac{{\left(x-\mu \right)}^{2}}{2\sigma^{2}}\right)$ Consequently, where

1. the mean age of adults over 25 years old = 48.9 years,

2. μ = 0.123 BMI change/year,

3. the BMI change/year above baseline BMI required for adult obesity (x) is ≥ 0.155 BMI change/year,

4. and the USA adult obesity rate (p(x)) = 33%;

the formula SD (σ) = 0.075. The SD of the FAO/WHO formula for female and male cohorts from all 167 countries was also derived from the normal distribution formula. Where

1. the mean age of adults over 25 years old = 38.9 years [29,32],

2. μ = 0.0720 BMI change/year (25.2 (mean adult BMI in 2008 for 334 cohorts) – 22.4 (baseline BMI) = 2.8; 2.8/38.9 years = 0.0720),

3. the BMI change/year above baseline required for adult obesity (x) is ≥ 0.155 BMI change/year, and

4. the weighted mean adult obesity rate in WHO countries (p(x)) = 14.1% [33];

the normal distribution formula yields an almost identical SD (σ) = 0.077 for the FAO/WHO formula.

To translate the FAO/WHO formula for mean adult BMI in the 167 countries to a FAO/WHO formula for BMI change/year, the SD was equated to 0.077 by multiplying the FAO/WHO mean adult BMI formula by 0.03882 (i.e., 1.984 (SD of FAO/WHO adult BMI prediction formula) * 0.03882 = 0.077). The b-weight of alcohol (g) in the FAO/WHO formula was doubled to compensate for alcohol not appearing in the DCCT/EDIC formula. With this adjustment for alcohol, the mean output of the weight impact of alcohol of the two formulas should better reflect the data. Finally, the output was centered at 0.07289 BMI change/year above baseline BMI per WHO data [17] by adding a constant. This gives the adjusted FAO/WHO formula below:

"BMI change/year FAO/WHO formula = (0.07710 alcohol (g) + 11.95 carbohydrates g * 4 (g/kcal)/kcals – 304.85 dietary fiber g/kcals + 19.7433 total fat g – 63.567 PUFA g * 9 (g/kcal)/kcals – 2.14356 exercise (DCCT 1–4 scale)) * 0.04115 – 0.05033; R^2^=0.54"

In order to synchronize the output of the two formulas, the DCCT/EDIC formula was adjusted to correspond with the FAO/WHO formula—i.e., the SD changed to 0.077 BMI change/year, and a constant added to change the mean output to 0.07289 BMI change/year above the baseline (BMI=22.4):

"Synchronized DCCT/EDIC BMI change/year formula = (0.898 protein g * 4/kcals + 1.063 carbohydrates g * 4 (g/kcal)/kcals – 13.19 dietary fiber g/kcals + 0.973 (total fat g – PUFA g) * 9 (g/kcal)/kcals – 0.04468 exercise (DCCT 1–4 scale) * 1.574 – 1.001;"

### Correlations of the FAO/WHO and DCCT/EDIC diet and exercise profiling formulas

The continuous model macronutrient and exercise formula for BMI change/year correlated strongly with the FAO/WHO categorical model food groups and exercise BMI change/year formula (r = 0.86, *P* < 0.0001). Testing with both the FAO/WHO and DCCT/EDIC datasets, the FAO/WHO and DCCT/EDIC macronutrient and exercise formulas also correlated with each other (r = 0.79, *P* < 0.0001 and r = 0.81, *P* < 0.0001, respectively, Tables 12 and 13).

**Table 12:** Correlations of FAO/WHO food group availability/macronutrient availability and exercise profiling formulas and DCCT/EDIC formula (n=334 cohorts)

**Outcome or formula**	**FAO/WHO mean BMI formula from food group availability and DCCT exercise r*(P)***	**FAO/WHO formula from macro-nutrient availability and DCCT exercise r*(P)***	**DCCT BMI change per year formula for macro-nutrient availability and exercise r (*P)***	**FAO/WHO and DCCT macro-nutrient formulas averaged r (*P)***
Mean BMI FAO/WHO countries	0.84 (<0.0001)	0.74 (<0.0001)	0.65 (<0.0001)	0.73 (<0.0001)
FAO/WHO mean BMI formula from food group availability and DCCT exercise (1–4 scale)	1.0	0.87 (<0.0001)	0.80 (<0.0001)	0.87 (<0.0001)
FAO/WHO mean BMI formula from macronutrient availability and DCCT exercise (1–4 scale)	0.87 (<0.0001)	1.0	0.87 (<0.0001)	0.98 (<0.0001)
DCCT BMI change/year formula for macronutrient availability and DCCT exercise (1–4 scale)	0.80 (<0.0001)	0.87 (<0.0001)	1.0	0.95 (<0.0001)

**Table 13:** Correlations of DCCT cohort (n=1,055) BMI change/year with profiling formulas

**Outcome or formula**	**BMI change/year DCCT cohort n = 1,055 r*(P)***	**FAO/WHO formula from macronutrient availability and DCCT exercise r*(P)***	**FAO/WHO and DCCT macronutrient formulas averaged r*(P)***
FAO/WHO mean BMI formula from macronutrient availability and DCCT exercise (1–4 scale)	0.15 (<0.0001)	1.0	0.98 (<0.0001)
DCCT BMI change/year formula for macronutrient availability and DCCT exercise (1–4 scale)	0.18 (<0.0001)	0.80 (<0.0001)	0.91 (<0.0001)
FAO/WHO and DCCT macro-nutrient formulas averaged	0.17 (<0.0001)	0.98 (<0.0001)	1.0

### Predicting weight impacts of individual foods and beverages

The FAO/WHO macronutrient (continuous model profiling) BMI change/year formula above with macronutrients expressed in g, the b-weight of alcohol doubled, and the exercise variable omitted is as follows:

"FAO/WHO adult cohort BMI change/year prediction = (0.07710 alcohol (g) + 11.95 carbohydrates g * 4 (g/kcal)/kcals – 304.86 dietary fiber g/kcals + 19.733 total fat g * 9 (g/kcal)/kcals – 63.567 PUFA g * 9 (g/kcal)/kcals)) * 0.04115 – 0.05033; R^2^=0.29"

As described in the methods, the above FAO/WHO adult BMI prediction formula was transformed into a weight impact of macronutrients formula (continuous profiling) for individual foods and beverages:

"FAO/WHO formula for four-year weight impact (pounds) = (0.07710 alcohol g + 11.95 (381.7 + carbohydrates g per serving) * 4 / (2,613 + kilocalories per serving) – 304.9 (30.38 + dietary fiber g per serving) / (2,613 + kilocalories per serving) + 19.73 (84.44 + total fat g per serving) * 9/(2,613 + kilocalories per serving) – 68.57 (20.45 + PUFA g per serving) * 9/(2,613 + kilocalories per serving) ) * 2.941 – 12.78 (n=334, R^2^=0.29, P < 0.0001)."

Likewise, the DCCT/EDIC formula for predicting weight impacts of individual foods and beverages is as follows:

"DCCT/EDIC formula for four-year weight impact (pounds) = (0.898 (102.2 + protein g per serving) * 4/(2,297 + kilocalories per serving) + 1.063 (264.2 + carbohydrates g per serving) * 4/(2,297 + kilocalories per serving) – 13.19 (24.29 + dietary fiber g per serving) /(2,297 + kilocalories per serving) + 0.973 (74.59 + (total fat g per serving – PUFA g per serving) * 9/(2,297 + kilocalories per serving)) * 85.82 – 68.12 (n=1,055, R^2^=0.03, P < 0.0001)."

Of note, kcals did not enter either formula. Independent of the macronutrient profile, calorie intake did not predict long term weight. The type of foods and beverages consumed rather than the number of kcals determined the long term weight impact.

Using data from the USDA National Nutrient Database for Standard Reference Release 24 [19], Table 14 details the kcals and macronutrients in average servings of 22 categories of foods and beverages reported by Mozaffarian and colleagues from the Harvard nutritional epidemiology team [15]. Table 15 shows the FAO/WHO and DCCT/EDIC formula predicted weight impacts of the 22 selected foods and beverages in pounds/4 years based on the macronutrient formula predictions compared with the Harvard nutritional epidemiology team food group profiling study data. Generally, for high carbohydrate foods and beverages, if the total carbohydrate/dietary fiber ratio is < 10, the item tended to reduce weight according to the FAO/WHO and DCCT/EDIC formula predictions (e.g., fruits, vegetables, and whole grains). For high fat foods (e.g., nuts, meat, and dairy), if the ratio of total fat/PUFA < 6, the FAO/WHO and DCCT/EDIC formulas predicted a lower weight in 4 years (e.g., nuts). For a total carbohydrate/dietary fiber ratio > 10 or a total fat/PUFA > 6, an increase in weight was predicted.

**Table 14:** **Nutrient composition from USDA National Nutrient Database for Standard Reference Release 24**[19]**of foods and beverages profiled by Mozaffarian and colleagues**[15]

**Dietary category**	**Serv-ing (g)**	**Kcals**	**Protein (g)**	**Total carbs (g)**	**Fiber (g)**	**PUFA (g)**	**MUFA (g)**	**SFA (g)**	**Total fat (g)**	**Alco-hol**
Fruits	154	68.5	0.86	15.8	2.25	0.16	0.60	0.15	1.04	0.00
Vegetables	116	40.8	2.62	7.33	2.39	0.26	0.11	0.09	0.58	0.00
Nuts	28	172	3.51	5.25	2.46	4.43	8.44	2.87	16.5	0.00
Whole grains	50	142	5.90	27.0	4.71	0.95	0.55	0.38	2.12	0.00
Refined grains	50	128	3.57	21.6	1.06	1.12	1.05	0.59	2.99	0.00
Sugary sodas	368	150	0.00	38.7	0.00	0.00	0.00	0.00	0.00	0.00
100% fruit juice	245	116	1.02	28.5	0.40	0.09	0.04	0.04	0.30	0.00
Non caloric soda water	360	0.00	0.00	0.00	0.00	0.00	0.00	0.00	0.00	0.00
Sweets or desserts	62	204	2.87	25.1	1.51	1.61	4.12	3.91	10.7	0.00
Unprocessed meats	86	283	20.5	0.00	0.00	1.63	9.16	8.37	22.0	0.00
Processed meats	41.3	341	18.0	2.21	0.00	2.69	13.2	10.1	28.4	0.00
Whole-fat dairy		104	6.67	3.15	0.00	0.25	1.90	4.35	7.24	0.00
Butter (1 tbsp)	15	108	0.12	0.00	0.00	0.45	3.33	7.65	12.2	0.00
Cheese	28	107	7.72	2.13	0.00	0.25	1.93	4.46	7.54	0.00
Whole milk	244	156	8.00	11.0	0.00	0.33	2.58	5.56	8.93	0.00
Low-fat dairy		96.6	7.59	12.2	0.00	0.07	0.54	1.21	1.92	0.00
Low-fat or skim milk	244	108	8.64	12.2	0.00	0.10	0.76	1.66	2.66	0.00
Yogurt	113	79.7	6.02	12.1	0.00	0.02	0.22	0.52	0.81	0.00
Potato chips	28	157	2.02	16.7	1.32	1.05	3.84	3.84	9.59	0.00
Potato with skin	173	171	4.08	39.3	3.90	0.09	0.00	0.04	0.19	0.00
Potatoes French fried	117	180	2.78	29.2	2.57	0.40	3.18	1.95	6.03	0.00
Alcohol (1 average drink of beer, wine, or spirits)	182	124	0.58	5.56	0.00	0.00	0.00	0.00	0.00	14.2

**Table 15:** Formula predicted change in pounds/4 years of selected foods based on the DCCT and FAO/WHO macronutrient intake analyses versus the Harvard diet lifestyle study data

**Dietary category**	**Kcals**	**Total carbs /fiber ratio**	**Total fats/PUFA ratio**	**FAO/WHO pounds change/4 years**	**DCCT pounds change/4 yrs**	**Average DCCT and FAO/WHO estimates**	**Harvard study pounds change /4 yrs**
Fruits	68.5	5.67	6.16	−0.15	−0.21	−0.18	−0.49
Vegetables	40.8	4.06	2.33	−0.68	−0.75	−0.71	−0.22
Nuts	172	3.06	3.51	−1.11	−0.98	−1.04	−0.57
Whole grains	142	6.43	3.12	−1.04	−1.00	−1.02	−0.37
Refined grains	128	27.7	3.35	0.01	0.20	0.09	0.39
Sugary beverages (12 oz)	150	∞	NA	1.27	1.60	1.44	1.00
100% fruit juice	116	50.0	3.80	0.79	1.05	0.92	0.31
Non caloric diet soda (12 oz)	0	NA	NA	0.00	0.00	0.00	−0.11
Sweets or desserts	204	37.1	15.8	0.79	0.51	0.65	0.41
Unprocessed meats	283	NA	13.4	0.75	0.61	0.68	0.95
Processed meats	341	35	10.8	0.71	0.37	0.54	0.93
Whole-fat dairy foods	104	∞	28.5	0.90	0.57	0.74	0.10
Butter (1 tbsp)	108	NA	27.1	1.53	0.63	1.08	0.30
Cheese	107	∞	30.2	0.89	0.56	0.73	0.02
Whole-fat milk	156	∞	18.8	1.32	1.00	1.16	−0.06
Low-fat dairy foods	96.6	∞	30.7	0.44	0.66	0.55	−0.05
Low-fat or skim milk	108	∞	27.4	0.50	0.68	0.59	0.06
Low-fat yogurt	79.7	∞	30.0	0.40	0.61	0.51	−0.82
Potato chips	157	13.5	7.27	0.81	0.38	0.60	1.69
Potato with skin	171	10.1	2.22	−0.08	−0.15	−0.12	0.57
Potatoes French fried	180	11.4	15.1	0.50	0.15	0.32	3.35
Alcohol (14.2 g)	124	∞	-	2.93	−2.57	0.18	0.41

Overall, the FAO/WHO and DCCT/EDIC formula predictions had no significant correlation with the food group profiling predictions or with each other (Table 16). Alcohol consumption in grams entered the FAO/WHO future BMI prediction formula, but not the DCCT/EDIC formula. Consequently, the FAO/WHO and DCCT/EDIC formula estimates for the four-year weight impact of one drink per day of alcohol (averaging macronutrient profiles of beer, wine, and spirits) were markedly different (i.e., FAO/WHO = + 2.93 pounds/4 years and DCCT/EDIC= − 2.57 pounds/4 years, Table 15). However, the average value of the two formulas corresponds with the Harvard food and beverage profiling estimate of the 4 year weight impact of 1 drink (i.e., 0.18 versus 0.41 pounds, Table 15). This single divergent data point causes the two formulas to have no significant overall correlation (r = 0.11, *P* = 0.64, Table 16). The mean FAO/WHO and DCCT/EDIC four-year weight impact estimates of beer, wine, and spirits are 0.58, 0.10, and −0.03, respectively.

**Table 16:** Correlations of FAO/WHO and DCCT/EDIC formulas and the Harvard categorical food profiling results for 22 foods and beverages

**Food profiling system**	**FAO/WHO formula r (*P*)**	**DCCT BMI formula r (*P*)**	**DCCT and FAO/WHO formulas averaged r (*P*)**
FAO/WHO mean BMI for countries formula adjusted for weight change/4 years	1.0	0.11 (0.64)	0.74 (<0.0001)
DCCT BMI change/year formula adjusted for weight change/4 years	0.11 (0.64)	1.0	0.74 (<0.0001)
Harvard diet and lifestyle food profiling scheme	0.29 (0.20)	0.16 (0.48)	0.30 (0.18)

The average of the FAO/WHO and DCCT/EDIC formula predictions correlated strongly with 12 food group profiling findings of Mozaffarian and colleagues (r = 0.85, *P* < 0.0001, Table 17). However, formula predictions trended towards a negative correlation with the Mozaffarian food group profiling findings for potatoes and dairy products (Table 18).

**Table 17:** Correlations of FAO/WHO and DCCT/EDIC formulas and 12 Harvard categorical food profiling results excluding potatoes and dairy products

**Food profiling system**	**FAO/WHO formula r (*P*)**	**DCCT/EDIC BMI formula r (*P*)**	**FAO/WHO and DCCT/EDIC formulas averaged r (*P*)**
FAO/WHO Mean BMI for countries adjusted for Weight change/4 years	1.0	0.03 (0.92)	0.70 (0.0115)
DCCT/EDIC BMI change/year formula adjusted for weight change/4 years	0.03 (0.92)	1.0	0.74 (0.0126)
Harvard Diet and Lifestyle Food Profiling Scheme	0.80 (0.0019)	0.57 (0.0507)	0.85 (<0.0004)

**Table 18:** Correlations of FAO/WHO and DCCT/EDIC macronutrient profiling formulas and the Harvard categorical food profiling results for 10 potato and dairy products

**Food profiling system**	**FAO/WHO formula r (*P*)**	**DCCT BMI formula r (*P*)**	**DCCT and FAO/WHO formulas averaged r (*P*)**
FAO/WHO mean BMI for countries adjusted for weight change/4 years	1.0	0.68 (0.0311)	0.95 (< 0.0001)
DCCT BMI change/year formula adjusted for weight change/4 years	0.68 (0.0311)	1.0	0.88 (0.0008)
Harvard diet and lifestyle food profiling scheme	−0.12 (0.73)	−0.56 (0.09)	−0.33 (0.36)

### Web based health tool utilizing the multiple regression derived formula

To allow individuals, health professionals, and nutrition researchers to assess and monitor diet and lifestyle patterns by means of the macronutrient and exercise profiling formulas from the FAO/WHO and the DCCT/EDIC, NR designed a simple-to-use web-based tool [24]. Predicated on sustaining the inputted macronutrient profile and physical activity pattern on average over time, future BMI predictions are made. This long-term BMI prediction tool requires little nutritional or computer expertise on part of the user.

## Discussion

These univariate and multivariate analyses support the thesis that disproportionate weight gain is due primarily to lack of exercise and excessive availability/consumption of foods in Table 3 with r > 0 and not enough availability/consumption of foods with r < 0. In Table 3, the r values of the breakdown of items under a broad food group heading probably have less significance that the r value of the broad heading. For instance, individual cereals vary significantly in r values (i.e., broad heading of cereals: r = −0.46, *P* < 0.0001, and subheadings: rice (r = −0.41, *P* < 0.0001), maize (r = −0.25, *P* < 0.0001), and wheat (r = 0.41, *P* < 0.0001)). This probably indicates that low BMI countries eat more rice and maize and high BMI countries eat more wheat, and much of the wheat in high BMI countries is likely refined into white (low fiber) flour. Overall in country populations, a high proportion of kcals as cereal contributes significantly to relatively lower mean BMIs.

It may be counterintuitive that fruit should be associated with excessive weight gain (i.e., fruit: r=0.22, P < 0.0001 in Table 3). Data from the diet and lifestyle study by Harvard nutritional epidemiologists showed that fruit consumption was associated with significantly decreased weight over a four year span while 100% fruit juices correlated with substantial weight gain (Table 15) [15]. In that study of over 120,000 USA participants, the mean intake of fruit juices was about half of the mean intake of fruit (0.73 juice servings/day versus 1.43 whole fruit servings per day). Under the FAO food group category, “FRUITS AND DERIVED PRODUCTS,” is the following explanation, “Fruit crops are consumed directly as food and are processed into dried fruit, fruit juice, canned fruit, frozen fruit, jam, alcoholic beverages, etc.” [34]. In the USA, US Department of Agriculture data show that about 40% of fruit availability is in the form of juices [35]. Consequently, whole unprocessed fruit likely correlates with normal BMIs while fruit juices likely correlate with overweight and obesity.

Based on both of these BMI change/year formulas, the adage, “eat less and exercise more” should be clarified to “eat more cereals, fruits, vegetables, pulses, roots, and tubers and exercise more” or “eat more high fiber carbs and more high PUFA fats and exercise more.”

In discussing the counterintuitive prediction that one serving per day of low-fat yogurt correlated with the largest weight loss of any food or beverage in their study (−0.82 pounds/4 years), Mozaffarian and the Harvard nutritional epidemiology team allowed for the possibility of “an unmeasured confounding factor that tracks with yogurt consumption” [15]. The paradox of low-fat yogurt associated with weight loss in the Harvard study while potato consumption correlated with increased weight may be due to confounding in three ways:

1. the association of dairy product consumption, particularly low-fat yogurt, with fruits, vegetables, nuts, and whole grains and with above average exercise in educated, relatively affluent, health-conscious people,

2. the association of potato consumption with oil or fat (e.g., butter, sour cream, etc.), and

3. the greater affordability and therefore consumption of inexpensive foods like French Fries, potato chips, sugar sweetened drinks, hamburgers, etc. for the lower socio-economic classes with higher rates of obesity.

Among the many organizations extolling the health benefits of low-fat yogurt are Cleveland Clinic [36], Mayo Clinic [37], Center for Science in the Public Interest [38], American Heart Association [39], FDA and USDA [40]. Due to these endorsements and the heavy advertising of dairy products; educated, health conscious, relatively more affluent people may respond by consuming more low-fat yogurt (and other dairy products) compared with less health conscious people that drink less expensive sugar sweetened beverages.

Data for analyzing the overall diet and exercise pattern associated with dairy foods consumption come from the “CARDIA Study,” a general community sample from four U.S. metropolitan areas [41]. CARDIA Study participants were partitioned into terciles according to consumption of dairy foods. Dairy consumption correlated with 18% more physical activity (the highest tercile in dairy consumption overall averaged about 18% more physical activity than the lowest dairy consuming tercile). Similarly, dietary profile comparisons of the highest and lowest dairy product consuming terciles showed that the highest tercile dairy consumers averaged 68% more whole grains, 13% more fruits and vegetables, and 46% less sugar-sweetened beverages than the tercile consuming the least dairy foods. Estimating conservatively, the highest dairy product tercile consumed at least 40% more dietary fiber/day (i.e., ≥ 10 g/day more) than the lowest. Using the FAO/WHO database, plugging these fiber and exercise values (i.e., 10 g/day more fiber and 18% more exercise) into the two formulas yielded an average prediction that the highest tercile of dairy consumers will gain 0.059 BMI units/year less than the lowest tercile (FAO/WHO formula: 0.065 BMI units/year less and DCCT/EDIC formula: 0.054 BMI units/year less). A similar formula calculation using the DCCT/EDIC database predicted that the highest tercile of dairy consumers will gain 0.069 BMI units/year less than the lowest tercile (FAO/WHO formula: 0.076 BMI units/year less and DCCT/EDIC formula: 0.063 BMI units/year less). An increase of 0.059 - 0.069 BMI units/year is in the range of overall development of the obesity epidemic (i.e., 2.95 – 3.45 extra BMI units in 50 years).

The National Health and Nutrition Examination Survey data show that average gains per year in BMI of the USA adult population ranges from 0.087 BMI units - 0.137 BMI units [42]. These data suggest that the relatively healthy overall diet and exercise pattern associated with low-fat yogurt may reduce long-term weight gain, but low-fat yogurt itself, and similarly other dairy products, more likely increase weight.

Rates of obesity in the U.S. and other developed countries are much higher in the food-insecure lower socio-economic classes [43]. Potatoes (including French Fries and potato chips), sugary foods, low PUFA meats, and refined grains provide dietary energy at the lowest cost and are chosen by poor people out of necessity [44]. As suggested by Drewnowski, food group (categorical) profiling studies linking inexpensive potatoes with obesity may be confounded because low income people, who carry higher risks of obesity, eat more potatoes [45]. FAO/WHO and DCCT/EDIC macronutrient profiling analyses (continuous model profiling) would not be subject to these kinds of biases related to food selections shaped by the income or health consciousness of the consumer.

FAO/WHO formula estimates for dairy products and potatoes accorded with DCCT/EDIC formula predictions (Table 18: n = 10, r = 0.68 *P* = 0.0311). Both macronutrient profiling formulas disagreed with the Harvard nutritional epidemiology team food group profiling estimates for dairy products and potatoes (r = −0.12, *P* = 0.73 and r = −0.56, *P* = 0.09 for the FAO/WHO and DCCT/EDIC versus Harvard weight impact predictions, respectively, Table 18). This supports the view that the health conscious public’s misperceptions of the long-term weight effect of low-fat yogurt and other dairy products and greater affordability of potatoes for low income people confounded the Harvard nutritional epidemiology team’s predictions concerning dairy products and potatoes.

While this analysis has potential confounders, it is, hopefully, a valuable first step with the methodology of comparing long-term data on BMI change/year to macronutrient and exercise profiles in different databases. Limitations of this study include: (1) food availability (FAO) is used rather than food consumption for the diet variables in countries, (2) only 167/200 FAO/WHO countries provided sufficient data on which to base an analysis, (3) imputed physical activity data was used for 55 female and 55 male cohorts, (4) data is lacking in the FAO database on nuts, seeds, and vegetables, (5) the DCCT/EDIC data included subjects with a relatively narrow range of variability in macronutrient intake and exercise level, (6) people with type 1 diabetes are not typical of the population for many reasons, so the univariate and multivariate correlations of DCCT/EDIC participants cannot be assumed to be the same as other populations, (7) the foregoing DCCT/EDIC factors probably led to a weak multiple variables correlation with BMI change/year (R^2^ = 0.03, P < 0.0001), (8) unequal access to various foods, cultural differences, and other factors may also confound the results of this analysis, and (9) these food group/macronutrient and exercise profiling formulas, although validated by the strong correlation with the food group profiling data from the Harvard nutritional epidemiology group, still require further verification from other databases relating macronutrient and exercise profiles to BMI change/year or adult BMI or from prospective diet and exercise profiling studies.

Inferring the changes in BMI based on the diet and physical activity parameters using the data from these studies may not be optimal, but it is reasonable given that the FAO kcal and macronutrient availability data for each entire country’s population would not be expected to change radically over 50 years for most countries. The exceptions will be part of the noise in the data. The DCCT diet analyses were conducted 2–5 times over the 4–9 years on trial and, unfortunately, not repeated during the EDIC 10 year follow-up. Ideally, we should have diet data on a yearly or monthly basis over decades. However, such data do not yet exist.

In countries with mean BMI levels already in overweight or obese categories or projected to increase into these categories, policymakers, nutrition professionals, and the public should consider that these formulas might inform strategies to combat the obesity epidemic. This should stimulate discussion about strategies to increase physical activity and adjust the availability and consumption of foods that increase BMI relative to BMI decreasing foods to avoid excessive weight gain and the associated health problems for individuals and populations.

Policymakers, dietary professionals, and individuals could also consider using or promoting the use of the website health tool offered in this article to base a “nudge” for people. According to the popular book, *Nudge: improving decisions about health, wealth, and happiness* by Richard Thaler and Cass Sunstein, the concept of nudging describes “any aspect of the choice architecture that alters people’s behavior in a predictable way without forbidding any options or significantly changing their economic incentives” [46]. Nudging uses “libertarian paternalism,” a political/social philosophy in which people’s choices are actively guided in their best interests but they remain at liberty to behave differently [47]. Regular analysis and monitoring of the long-term weight impacts of one’s diet and physical activity choices with this tool could nudge people to adopt healthier lifestyles in accordance with their own perceived best interests.

The correlations between these two formulas and the validation of the formulas by comparison with the food group profiling data of the three databases used by Mozaffarian and colleagues raise the possibility that multiple regression formulas derived from all other databases may also have a similar format:

"Possible general format for BMI change/year prediction formulas = (A * carbohydrates g * 4 (g/kcal)/kcals – B * dietary fiber g/kcals + C * total fat g * 9 (g/kcal)/kcals – D * PUFA g * 9 (g/kcal)/kcals – E * exercise) * F + G;"

While protein and alcohol were each only in one formula, the univariate correlations of both of these macronutrients in the FAO/WHO database suggest that they tend to increase weight (i.e., r > 0, Table 8). The statistical findings of this analysis support previous recommendations to encourage consumption of mostly unprocessed plant-based commodities (fruits, vegetables, cereals, pulses, roots/tubers, etc.) to combat the obesity epidemic. The formulas in this study may facilitate strategies by individuals and policy makers to nudge the patterns of food consumption in a healthy direction. Further, immediate feedback on the predicted long-term effect of exercise from the health tool should be combined with strategies to promote regular physical activity at population levels (e.g., in schools, worksites, etc.) and to incentivize regular physical activity in the health care system.

Utilizing the website tool offered in this article could provide a welcome “nudge” to motivated users to adopt diet and exercise habits in line with their wishes for long-term weight control. Academic nutrition researchers should consider partnering with the authors in undertaking prospective observational/interventional studies of individuals that use the future BMI prediction interactive website to prevent or treat obesity.

## Abbreviations

*: Multiplied by; BMI: Body mass index; carbs: Carbohydrates; DCCT: Diabetes Control and Complications Trial; EDIC: Epidemiology Diabetes Intervention and Complications; FAO: Food and Agriculture Organization; g: Grams; HbA1c: Hemoglobin A 1c; Kcals-Kilocalories: kg/m^2^, Kilograms per meter squared; PUFA: Polyunsaturated fatty acids; MUFA: Monounsaturated fatty acids; R^2^: Coefficient of multiple correlations; r: Coefficient of correlation; SD (σ): Standard deviation; SFA: Saturated fatty acids; USDA: United States Department of Agriculture; WHO: World Health Organization.

## Competing interests

This analysis was not supported by public or private funding. DKC and NR have no conflicts of interest. They have completed the Unified Competing Interest form at http://www.icmje.org/coi_disclosure.pdf (available on request from the corresponding author) and declares that (1) DKC and NR have received no financial support for this article; (2) DKC and NR have no relationships that might have an interest in the submitted work in the previous three-years; (3) their partners or children have no financial relationships that may be relevant to the submitted work; and (4) DKC and NR have no non-financial interests that may be relevant to the submitted work.

## Authors’ contributions

DKC obtained the DCCT/EDIC data sets from the National Technical Information Service in collaboration with the University of Pittsburgh Endocrinology Department and the FAO/WHO databases from the Internet. He wrote the statistical investigation plan, cleaned and analyzed the data, and drafted and revised the paper. He had full access to all of the data in the study and takes responsibility as guarantor for the integrity of the data and the accuracy of the data analysis. NR designed an interactive website termed a “health tool” to allow the public and healthcare professionals to analyze the long-term BMI change/year impact of individual diet and lifestyle patterns according to these FAO/WHO and DCCT/EDIC data derived formulas. He also critiqued the paper and drafted the narrative description of the web-based health tool. All authors read and approved the final manuscript.
